# Seasonal Dynamics of Aquatic Eukaryotic Communities in a Yangtze Delta Shallow Drinking-Water Reservoir Revealed by 18S rRNA Gene Metabarcoding

**DOI:** 10.3390/biology15212036

**Published:** 2026-11-08

**Authors:** He Zhang, Xudong Wang, Zhenfeng Tu, Cheng Feng, Yiqi Li, Peng Xiao, Qihang Zhao, Jun Zuo, Yaqiong Lan, Renhui Li

**Affiliations:** 1 Zhejiang Provincial Key Laboratory for Subtropical Water Environment and Marine Biological Resources Protection, National and Local Joint Engineering Research Center of Ecological Treatment Technology for Urban Water Pollution, College of Life and Environmental Sciences, Wenzhou University, Wenzhou 325035, China; zhanghe1983004@sina.com (H.Z.);; 2 Department of Environment, Yangtze Delta Region Institute of Tsinghua University, Jiaxing 314006, China; wangxudong3132@163.com; 3 Wenzhou Water Supply Co., Ltd., Wenzhou 325000, China; 4 The Ecological Environment Bureau of Jiaxing Haiyan, Jiaxing 314300, China; 5 State Key Laboratory of Lake Science and Environment, Nanjing Institute of Geography and Limnology, Chinese Academy of Sciences, Nanjing 210008, China

**Keywords:** plain shallow drinking-water reservoirs, aquatic eukaryotic communities, 18S rRNA, seasonal dynamics, ASV turnover, environmental associations

## Abstract

Aquatic eukaryotic communities play important roles in biogeochemical nutrient cycling and ecosystem functioning and are also sensitive to changes in aquatic environmental conditions. Qianmudang Reservoir is a typical shallow drinking-water reservoir supplying approximately 500,000 residents in Haiyan County, Zhejiang Province, China. However, the seasonal dynamics of its aquatic eukaryotic communities remain poorly understood. In the present study, we used 18S rRNA gene amplicon sequencing to characterize aquatic eukaryotic communities across four seasonal surveys in 2025. A total of 4903 eukaryotic amplicon sequence variants (ASVs) were obtained. ASV richness and Shannon diversity were highest in summer. *Cryptomonas* (Cryptophyta) showed the highest overall relative sequence abundance at the genus level, TSAR accounted for the greatest relative sequence abundance at the supergroup level. Community composition showed pronounced seasonal differentiation, and indicator value analysis identified 17 season-associated genera. Only 33 ASVs were detected across all four seasons, and β-diversity partitioning revealed that seasonal community dissimilarity was dominated by ASV turnover rather than nestedness-resultant differences. Partial db-RDA identified associations between community composition and total phosphorus, dissolved oxygen and water temperature, where total phosphorus had the strongest marginal effect. Variation partitioning further showed that environmental variables and sampling period jointly explained a large proportion of community variation. These findings revealed pronounced seasonal restructuring of aquatic eukaryotic communities at both taxonomic and ASV levels and highlight the importance of ASV turnover and occupancy patterns for understanding temporal biodiversity dynamics in shallow drinking-water reservoirs.

## Simple Summary

Shallow drinking-water reservoirs are vital for regional water supply, yet the seasonal patterns of aquatic eukaryotes, which underpin reservoir ecosystem function, remain poorly understood. In this study, we used 18S rRNA gene metabarcoding to survey aquatic eukaryotic communities across four seasons in Qianmudang Reservoir, a plain shallow reservoir supplying drinking water for about half-million residents. We found that distinct seasonal shifts in eukaryotic assemblages, with the highest ASV richness and Shannon diversity occurring in summer. Only very few ASVs persisted year-round across all four seasons, and community seasonal dissimilarity was mainly driven by ASV turnover rather than nestedness. Partial db-RDA identified significant associations with total phosphorus, dissolved oxygen and water temperature. Variation partitioning further revealed strong shared explanatory effects between environmental variables and sampling period. Our findings underline the importance of ASV-level turnover patterns and seasonal indicator genera for biological monitoring and ecosystem management of shallow drinking-water reservoirs.

## 1. Introduction

Lakes and reservoirs are critical components of centralized drinking water supplies in China, and their role in ensuring water security is increasingly vital, especially in the densely populated eastern regions [1,2]. However, shallow drinking water sources in the Yangtze River Delta plain have long faced various ecological challenges, such as algal blooms and taste-and-odor problems [3,4]. Additionally, many lakes and reservoirs exhibit significant seasonal fluctuations in physicochemical conditions, including water temperature (WT), dissolved oxygen content (DO), transparency, nutrient concentrations, and microbial community structure [5,6,7]. As a result, investigations conducted at a single time point or location may not adequately capture the annual ecological dynamics of aquatic eukaryotic communities in reservoirs [8].

Furthermore, the factors that influence planktonic eukaryotic community structures differ among various aquatic ecosystems. For instance, Huang et al. conducted a year-round survey of Dongqian Lake and characterized eukaryotic communities by 18S ribosomal RNA (18S rRNA) gene amplicon sequencing [5]. They found that the communities exhibited greater similarity between summer and autumn, whereas substantial differences were noted between spring and winter. Notably, seasonal variation had a more pronounced effect on community composition than spatial differences among sampling sites [5]. Similarly, Zhang et al.’s four-season study of Lake Chaohu and its seven major inflowing rivers revealed significant seasonal variations in aquatic eukaryotic community structure, with the highest α-diversity observed in summer. They noted that WT, DO, and total phosphorus (TP) content were significantly associated with community composition [9]. These findings indicate that seasonal environmental fluctuations continuously reshape aquatic eukaryotic communities. Therefore, multi-seasonal monitoring is essential for accurately characterizing ecological dynamics in shallow drinking water sources and for providing a comprehensive basis for reservoir management and water quality regulation.

Aquatic eukaryotes encompass diverse functional groups, including algae, protists, fungi, and small metazoans [10]. Algae serve as primary producers, whereas protists and small metazoans contribute to predation processes and energy transfer within aquatic food webs [11,12,13]. Fungi, in contrast, play important roles in organic matter decomposition and parasitic interactions [14]. These taxonomic groups differ substantially in their ecological functions, life-history strategies, and environmental sensitivities. Therefore, monitoring a single group alone does not provide a comprehensive understanding of how aquatic ecosystems respond to environmental changes [10]. Traditional morphological surveys have limited capacity for identifying taxa that are microscopic, morphologically similar, low in abundance, or characterized by complex life-history stages. In contrast, 18S rRNA gene amplicon sequencing enables simultaneous characterization of multiple eukaryotic lineages from a single water sample, thereby providing extensive information for a more comprehensive understanding of aquatic eukaryotic community structure [11,15,16]. Therefore, integrating environmental DNA-based high-throughput sequencing approaches to incorporate producers, consumers, and decomposers into a unified community analysis framework represents an essential strategy for improving the comprehensiveness of biological monitoring in shallow drinking water source systems.

Current studies of shallow aquatic ecosystems have primarily characterized eukaryotic community variations based on α-diversity, dominant taxonomic composition, and environmental associations [5,9]. However, these metrics are insufficient for determining whether observed community differences arise from abundance fluctuations in a limited number of taxa or from the appearance, disappearance, and replacement of numerous amplicon sequence variants (ASVs). Garner et al. conducted an 18S rRNA gene amplicon study across 366 freshwater lakes and found that most ASVs were restricted to a single lake or a small number of lakes, whereas widely distributed ASVs represented only a small proportion of the total ASV pool [17]. They reported that community turnover was primarily driven by environmental gradients, including nutrient availability, chlorophyll *a* (Chl-*a*) concentrations, WT, and watershed land use. These findings suggest that comparisons based solely on diversity metrics and higher-level taxonomic composition are inadequate for elucidating the ASV-level patterns that underlie aquatic eukaryotic community restructuring. Instead, a comprehensive assessment should integrate ASV-level characteristics, including ASV sharing patterns, ASV turnover, nestedness, sample occupancy, and their relative sequence contributions. However, seasonal studies of shallow drinking-water reservoirs rarely resolve community restructuring at the ASV level by jointly examining temporal sharing, turnover, nestedness, and occupancy-abundance relationships.

China’s Qianmudang Reservoir is a typical shallow drinking water source system in the Yangtze River Delta plain region. This water body has previously experienced disturbances in its microbial community structure and severe algal blooms. However, the seasonal dynamics of aquatic eukaryotic communities based on 18S rRNA gene amplicon sequencing data remain poorly understood. In particular, little is known about ASV turnover, occupancy patterns, and environmental associations. To address these knowledge gaps, we established five fixed sampling sites and conducted seasonal surveys during winter, spring, summer, and autumn of 2025. Specifically, the goals of this study were to investigate: (1) seasonal variations in physicochemical and community α-diversity; (2) seasonal differentiation of community composition and indicator genera; (3) ASV sharing patterns, ASV turnover, and occupancy-abundance relationships; and (4) the marginal association strength between individual environmental factors and community composition, accounting for spatial differences among sampling sites. The results of this study provide new insights into the seasonal ecological dynamics of aquatic eukaryotic communities in shallow drinking water sources and offer a scientific basis for biological monitoring and the identification of ecosystem responses to environmental changes.

## 2. Materials and Methods

### 2.1. Study Area, Sampling Design, and Environmental Measurements

**Figure 1:**
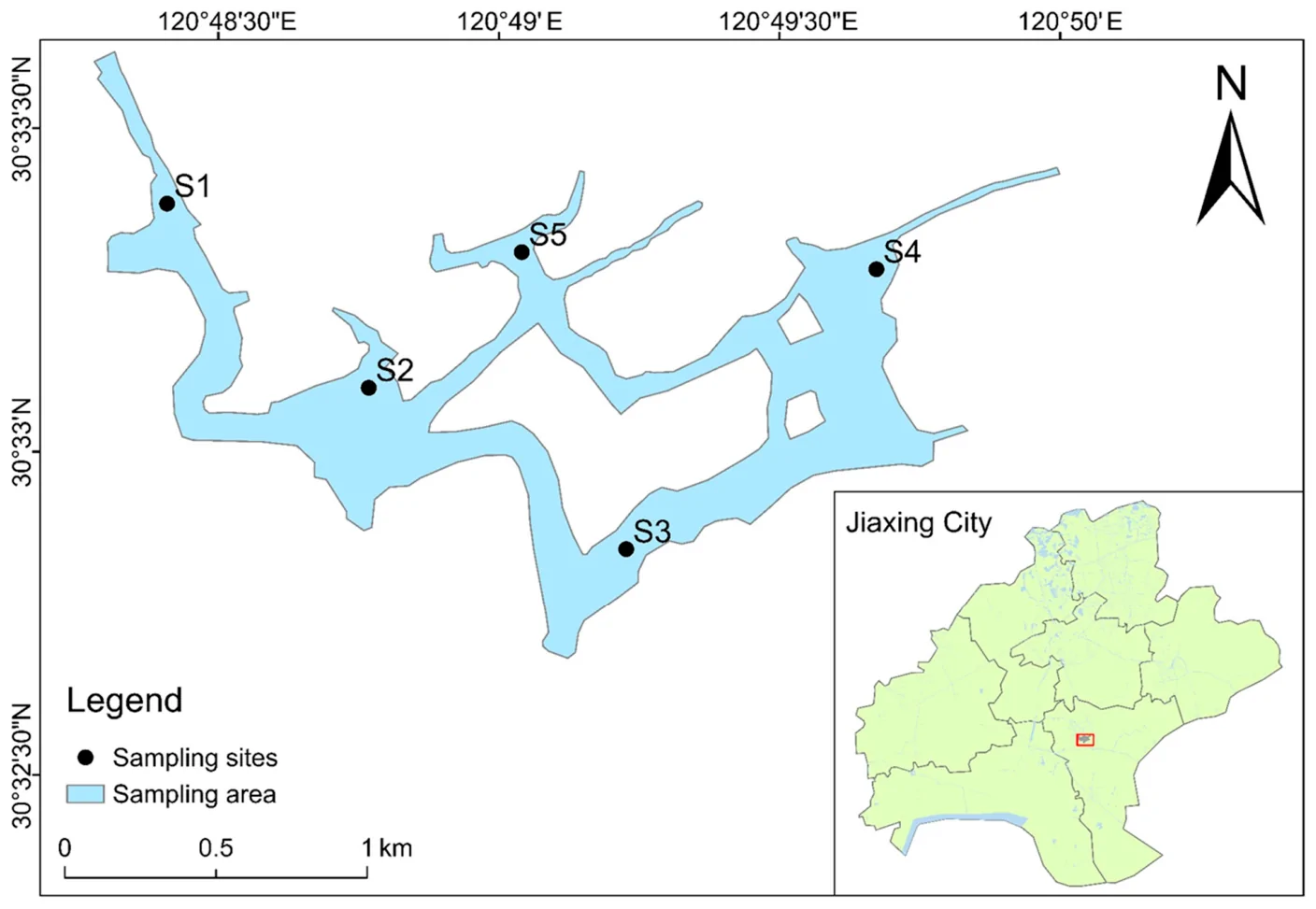
Map showing the locations of sampling sites in Qianmudang Reservoir.

Qianmudang Reservoir is situated in Haiyan County, Jiaxing City, Zhejiang Province, China. It has an average water depth of 3 m and a total storage capacity of 4.4 × 10^6^ m^3^. The water body exhibits a dendritic branching morphology and currently supplies drinking water to approximately 500,000 residents, making it one of the key shallow drinking water sources in the Yangtze River Delta plain region. The sampling area spans a longitude range of 120°48′30″–120°50′00″ E and a latitude range of 30°32′30″–30°33′30″ N. Five fixed sampling sites, designated as S1–S5, were established within the study area (Figure 1). Field surveys were conducted on 16 January, 23 April, 24 July, and 22 October, 2025, representing winter, spring, summer, and autumn, respectively. Surface water samples (5 L) were collected at 0.5 m below the water surface using a Kemmerer water sampler (Rongtong, Suzhou, China), and these field samples were pretreated within 12 h.

In situ measurements of water quality and physicochemical parameters were conducted following established protocols from previous studies [7,18] and standard water quality analysis methods [19]. Specifically, WT, DO, and pH levels were recorded in situ utilizing a portable multi-parameter water quality meter (Xylem EXO2, Ohio, USA). Secchi depth and water depth (WD) were assessed using a Secchi disk (diameter: 20 cm, black and white) and a single-beam echo sounder, respectively. In the laboratory, water samples were analyzed for content of total nitrogen (TN), TP, nitrate nitrogen (NO_3_^−^-N), ammonium nitrogen (NH_4_^+^-N), permanganate index (COD_Mn_), and phosphate (PO_4_^3–^-P). Chl-*a* concentrations were quantified using the ethanol spectrophotometric method [18].

### 2.2. DNA Extraction, 18S rRNA Gene Amplicon Sequencing, and Bioinformatics

DNA extraction, high-throughput sequencing, and bioinformatic analyses were conducted following established protocols [18,20,21]. In brief, approximately 500 mL of surface water from each sampling site was filtered using 0.22 μm GTTP membrane filters (Millipore, Billerica, MA, USA). After filtration, the filters were transferred into sterile cryogenic tubes and stored at −80 °C until used for DNA extraction. Total DNA was extracted utilizing the FastDNA Spin Kit (MP Biomedicals, Santa Ana, CA, USA) in accordance with the manufacturer’s instructions. The integrity of the extracted DNA was assessed through electrophoresis on a 1.0% agarose gel, and DNA concentration and purity were quantified using a NanoDrop 2000 spectrophotometer (Thermo Fisher Scientific, Waltham, MA, USA). The V4 region of the 18S rRNA gene was selected as the target amplification region and was amplified using the universal primers TAReuk454FWD1 (5′-CCAGCASCYGCGGTAATTCC-3′) and TAReukREV3R (5′-ACTTTCGTTCTTGATYRA-3′) [22,23]. The primers were synthesized by Tsingke Biotechnology Co., Ltd. (Beijing, China). PCR amplification was performed according to previously described protocols [21]. Briefly, each PCR reaction was conducted in a total volume of 100 μL, consisting of 50 μL of Phusion^®^ High-Fidelity PCR Master Mix (Vazyme, Nanjing, China), 2 μL each of the forward and reverse primers, 10 μL of genomic DNA template (1 ng/μL; 10 ng in total), and 36 μL of dd H_2_O. The PCR amplification program included initial denaturation at 98 °C for 1 min, followed by 30 cycles of denaturation at 98 °C for 10 s, annealing at 50 °C for 30 s, and extension at 72 °C for 30 s, concluding with a final extension step at 72 °C for 5 min. After assessing the quality of the PCR products, sequencing libraries were constructed using the NEBNext Ultra II DNA Library Prep Kit (New England Biolabs, Ipswich, MA, USA). The libraries were subsequently sequenced on an Illumina NovaSeq 6000 platform (Illumina, San Diego, CA, USA) using paired-end 250-base pair (PE250) sequencing [21,22].

Sequence processing was conducted in accordance with previously established protocols [24,25,26]. In brief, raw paired-end reads were processed using fastp for adapter trimming, filtering of low-quality bases, and removal of abnormally short reads. The processed sequences were subsequently imported into the Quantitative Insights Into Microbial Ecology 2 (QIIME 2) platform, where DADA2 was utilized for quality filtering, paired-end merging, error correction, chimera removal, and ASV inference. Representative ASV sequences were taxonomically assigned using the Protist Ribosomal Reference (PR2) database (version 5.0.0) [27]. For genus-level analyses, only taxa with a taxonomic confidence threshold of ≥0.70 and a clearly assigned genus name were retained. Database placeholder names and taxa that could not be reliably classified to the genus level were excluded from genus-level ranking and seasonal indicator genus analyses. To mitigate the effects of unequal sequencing depth among samples on α-diversity and taxonomic composition analyses, all samples were rarefied to the lowest effective sequencing depth of 58,954 sequences per sample.

### 2.3. Community Diversity, Composition, and Temporal Turnover Analyses

Based on the normalized ASV table, community richness, Shannon diversity index, Simpson diversity index, and Pielou’s evenness index were calculated to characterize patterns of community richness, diversity, and evenness [18,28,29]. Taxonomic composition was characterized using the relative sequence abundance of ASVs, which were taxonomically annotated and confidence-filtered prior to aggregation at the supergroup level. The four dominant supergroups—TSAR, Obazoa, Archaeplastida, and Cryptista—were visualized individually, while low-abundance supergroups and sequences lacking reliable supergroup-level taxonomic assignments were pooled into the “others” category.

For genus-level heatmap visualization, genera detected in at least five samples were retained, and the top 25 most abundant genera were selected based on their mean relative sequence abundance across all samples. Subsequently, row-wise Z-score normalization was applied to each genus to facilitate the comparison of its relative variation among different samples. To complement the genus-level comparison, mean relative sequence abundance was also summarized at the Division level using the same 20 samples (Figure S1).

Community compositional dissimilarity analysis was conducted using the raw ASV count matrix. ASVs detected in at least two samples and with a total sequence count of 20 or more were retained. A pseudocount of 0.5 was added to the filtered count matrix, followed by a centered log-ratio (CLR) transformation. Euclidean distances calculated from the CLR-transformed data were utilized as Aitchison distances [30]. Subsequently, principal coordinates analysis (PCoA) was performed to visualize the differences in community composition among the samples.

An ASV was deemed present during a specific sampling period if it was detected in at least one of the five sampling sites within that time frame. An UpSet plot was used to visualize the intersection patterns of ASVs across different sampling periods [31]. Utilizing the ASV presence–absence matrix, paired comparisons were performed between various sampling periods at the same sites. The overall community dissimilarity was quantified using the Sørensen dissimilarity index (βSOR), which was subsequently partitioned into the Simpson turnover component (βSIM) and the nestedness-resultant component (βSNE), as expressed by the equation βSOR = βSIM + βSNE [32,33].

Based on the number of sampling periods during which each ASV was detected, the ASVs were categorized into four groups: single-period ASVs, two-period shared ASVs, three-period shared ASVs, and four-period shared ASVs. The relative contributions of each ASV category to the sequence data were calculated for each sample. The sample occupancy of each ASV was defined as the number of samples in which the ASV was detected out of a total of 20 samples. The mean relative sequence abundance of each ASV was calculated as the average relative sequence abundance across the samples in which the ASV was present.

To evaluate the potential influence of metazoan sequences on our main findings, all analyses were repeated after removing ASVs taxonomically assigned to Metazoa (annotation confidence ≥ 0.70). Detailed methods and corresponding results are provided in the Supplementary Materials.

### 2.4. Statistical Analysis

Environmental variables and α-diversity indices underwent outlier detection, normality testing, and homogeneity of variance testing prior to comparisons among sampling periods. Variables meeting the assumptions of normality and homogeneity of variance were analysed using one-way analysis of variance (ANOVA) to test for differences across the four sampling periods, with Tukey’s Honestly Significant Difference test applied for post hoc pairwise comparisons [34]. For variables that did not meet the assumptions of parametric tests, the Kruskal–Wallis test was employed, followed by Dunn’s test for pairwise comparisons. *p*-values from multiple comparisons were adjusted using the Benjamini–Hochberg method, with statistical significance defined as *p* < 0.05.

Permutational multivariate analysis of variance (PERMANOVA) was conducted to evaluate the effects of both sampling season and sampling site on community composition [35]. In testing the effect of the sampling period, the sampling site was included as a covariate in the model, with permutations restricted to the same sampling site. Conversely, when examining the effect of the sampling site, the sampling period was included as a covariate, and permutations were restricted to the same sampling period. All PERMANOVA tests utilized 9999 restricted permutations. Additionally, permutational multivariate analysis of variance (PERMDISP) was applied to assess whether dispersion of community composition varied across sampling periods [35].

Indicator value (IndVal) analysis was performed to identify genus-level taxa associated with different sampling periods [36]. To be included in this analysis, genera had to be detected in at least five samples and to exhibit an overall mean relative sequence abundance of ≥0.05%. Statistical significance was evaluated using 9999 within-site restricted permutations, with *p*-values adjusted via the Benjamini–Hochberg method. For visualization in the main figures, only genera with adjusted *p* < 0.05 and an overall mean relative sequence abundance of ≥0.20% were retained. The relationship between ASV sample occupancy and the mean relative sequence abundance of each ASV when detected was assessed using Spearman’s rank correlation analysis.

Prior to environmental association analysis, variance inflation factor (VIF) analysis was conducted to evaluate multicollinearity across the 12 environmental variables [37]. All environmental variables were standardized prior to analysis. At each iteration, the variable with the highest VIF was removed when its VIF ≥ 5, and VIF values were recalculated for the remaining predictors. This iterative procedure continued until all retained variables exhibited VIF values < 5. Following this screening, NO_3_^−^-N, SD, PO_4_^3−^-P, TN, and Chl-*a* were sequentially excluded. The seven retained variables (TP, NH_4_^+^-N, COD_Mn_, WT, DO, pH, and WD) had final VIFs ranging from 1.49 to 3.66 and were used for downstream analyses. Notably, these exclusions aimed to mitigate multicollinearity and do not imply that the removed variables lack ecological relevance.

Using the CLR-transformed community matrix and Aitchison distance, partial distance-based redundancy analysis (partial db-RDA) was conducted to evaluate the relationships between environmental variables and community composition, with the sampling site included as a conditioning variable [35,38]. The overall model and the marginal effects of individual environmental variables were assessed using 9999 within-site restricted permutations. The overall model was reported using adjusted R^2^, pseudo-F statistics, and *p*-values, while the strength of association for individual environmental variables was quantified using marginal pseudo-F. All *p*-values were adjusted using the Benjamini–Hochberg correction. To further distinguish seasonal and environmental effects, variation partitioning analysis (VPA) was conducted with sampling period and the seven preselected environmental variables defined as two separate explanatory matrices. Adjusted R^2^ values were used to quantify their independent and shared contributions to community variation [39].

The sampling site distribution map was generated using ArcMap 10.8.1. Sequence processing was performed on the QIIME 2 platform [25], and community ecological analyses and statistical visualizations were primarily conducted using R version 4.3.2. The vegan package facilitated α-diversity analysis, PCoA, PERMANOVA, PERMDISP, and partial db-RDA analyses [35]. The indicspecies package was utilized for IndVal analysis, and the betapart package was employed for β-diversity partitioning [33,36]. Data visualization and figure assembly were accomplished using the ggplot2 and patchwork packages [40,41]. Unless otherwise specified, statistical significance was defined as *p* < 0.05.

## 3. Results

### 3.1. Spatial Setting and Seasonal Variation in Physicochemical Properties

**Figure 2:**
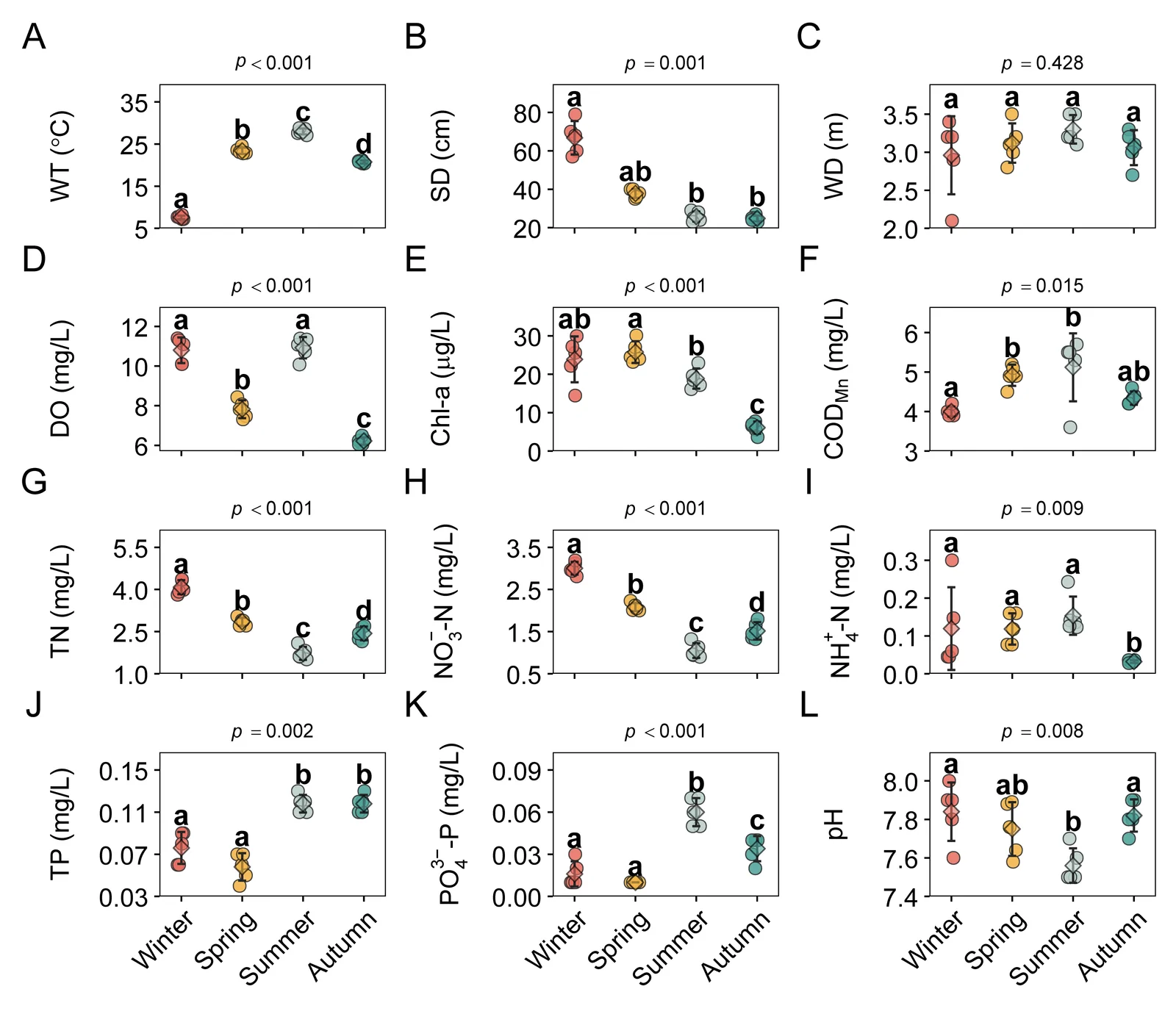
Seasonal variations in environmental variables in Qianmudang Reservoir. (**A**) Water temperature (WT); (**B**) Secchi depth (SD); (**C**) water depth (WD); (**D**) dissolved oxygen (DO); (**E**) chlorophyll *a* (Chl−*a*); (**F**) permanganate index (COD_Mn_); (**G**) total nitrogen (TN); (**H**) nitrate nitrogen (NO_3_^−^−N); (**I**) ammonium nitrogen (NH_4_^+^−N); (**J**) total phosphorus (TP); (**K**) phosphate phosphorus (PO_4_^3–^−P); and (**L**) pH. Dots denote individual site observations, diamonds show mean values, and error bars indicate standard deviations (*n* = 5). The *p*-value in each panel corresponds to the unadjusted global *p*-value derived from one-way ANOVA or the Kruskal–Wallis test. Different lowercase letters denote significant seasonal differences (*p* < 0.05). Groups sharing any common letter are not statistically distinct, while groups with no overlapping letters differ significantly (*p* < 0.05). Full statistical outputs are available in Table S1.

During the four sampling periods, environmental variables exhibited marked seasonal variations (Figure 2, Table S1). WT peaked in summer at 28.00 ± 0.70 °C and reached its lowest value in winter at 7.60 ± 0.42 °C. Significant differences were detected among the four seasons (Figure 2A). Secchi depth decreased from 66.8 ± 8.7 cm in winter to approximately 25 cm in summer and autumn. Winter displayed significantly higher transparency compared to summer and autumn, whereas WD did not show significant seasonal differences (Figure 2B,C). DO concentrations were relatively high in both winter and summer, reaching 10.80 ± 0.65 mg/L and 10.92 ± 0.54 mg/L, respectively, while the value declined to the lowest level in autumn (6.22 ± 0.18 mg/L) (Figure 2D). Chl−*a* concentrations peaked in spring at 25.78 ± 2.82 μg/L, reached their lowest level in autumn at 6.10 ± 1.60 μg/L, and showed intermediate values in winter and summer (Figure 2E). COD_Mn_ was lowest in winter (4.00 ± 0.12 mg/L), increased significantly in spring and summer, and exhibited intermediate values in autumn (Figure 2F).

Nitrogen and phosphorus nutrients exhibited distinct seasonal variation patterns. TN and NO_3_^−^−N levels peaked during winter, with concentrations of 4.08 ± 0.25 mg/L and 3.00 ± 0.15 mg/L, respectively. Both parameters declined to their lowest levels in summer (1.73 ± 0.23 mg/L and 1.05 ± 0.17 mg/L, respectively) and subsequently increased in autumn, with significant differences noted among the four seasons (Figure 2G,H). NH_4_^+^−N content did not exhibit significant differences among winter, spring, and summer, whereas it was significantly lower in autumn at 0.032 ± 0.004 mg/L (Figure 2I).

In contrast to nitrogen nutrients, TP in Qianmudang Reservoir reached 0.118 mg/L in both summer and autumn, and the values were significantly higher than levels recorded in winter and spring. The highest concentration of PO_4_^3–^−P occurred in summer (0.06 ± 0.01 mg/L), followed by autumn, while lower concentrations were observed in winter and spring (Figure 2J,K). The overall pH remained relatively stable at 7.74 ± 0.16, but summer values were slightly lower than those in winter and autumn, while spring exhibited intermediate values (Figure 2L).

### 3.2. Seasonal Variation in α-Diversity

**Figure 3:**
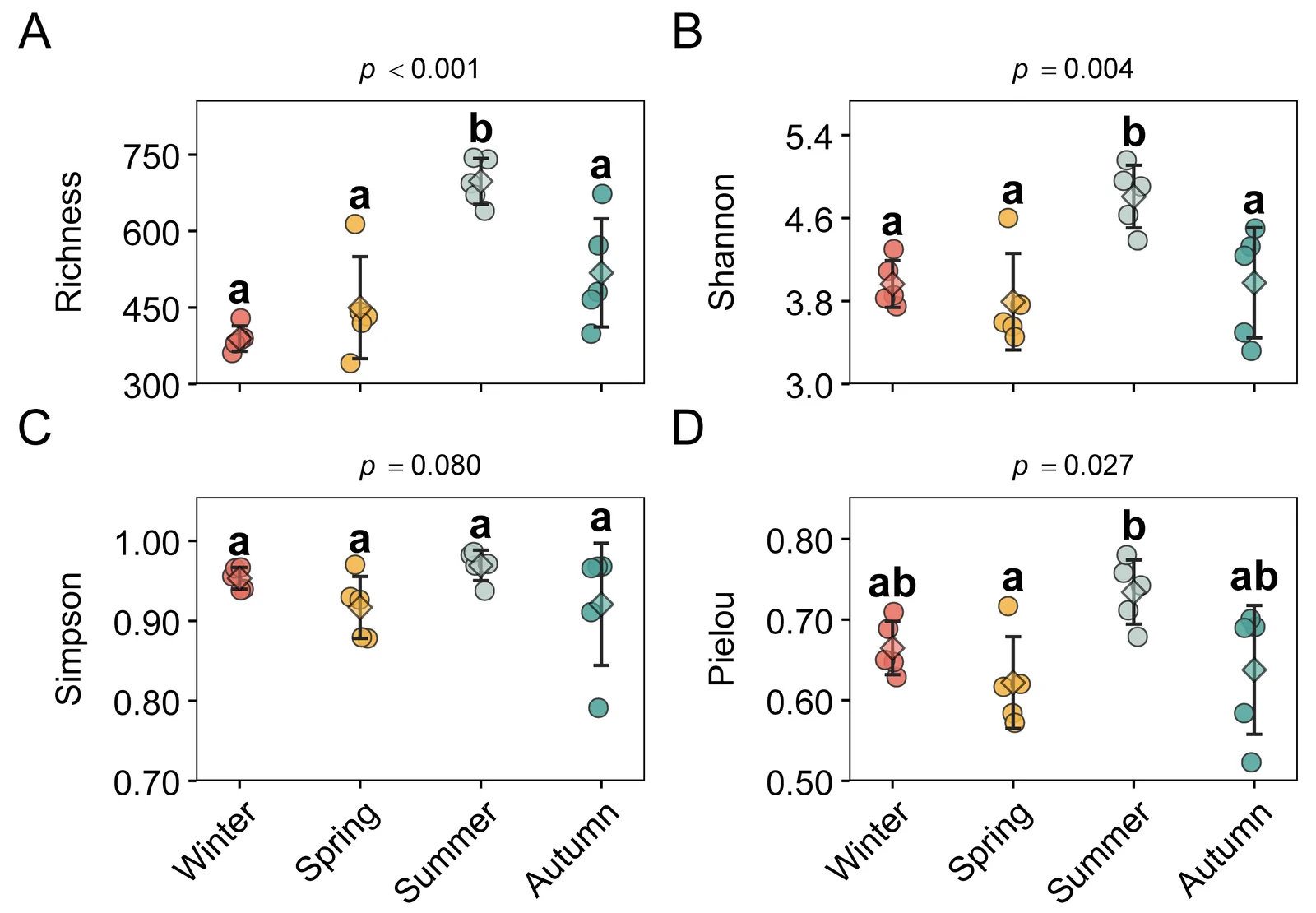
Seasonal variations in α-diversity indices of aquatic eukaryotic communities. (**A**) Richness; (**B**) Shannon index; (**C**) Simpson index; and (**D**) Pielou’s evenness index. Dots denote observations from individual sampling sites, diamonds show mean values, and error bars represent standard deviations (*n* = 5). The *p*-value in each panel is the unadjusted global *p*-value derived from one-way ANOVA or the Kruskal–Wallis test. Distinct lowercase letters mark significant seasonal differences (*p* < 0.05). Groups sharing at least one common letter are not statistically different, while groups with no overlapping letters differ significantly (*p* < 0.05). Full statistical results are provided in Table S2.

A total of 4903 ASVs were detected across the four sampling periods prior to rarefaction. After rarefaction, 19 low-abundance ASVs were eliminated from all samples, yielding 4884 ASVs with non-zero read counts. Using the rarefied ASV table, summer harboured the greatest ASV richness (1882), followed by autumn (1465), spring (1244), and winter (1107). The ASV composition in Qianmudang Reservoir exhibited clear seasonal variation, with α-diversity indices demonstrating varying degrees of seasonal variation (Figure 3, Table S2). ASV richness peaked in summer (698.0 ± 44.9), and the value was significantly higher than that in winter (389.4 ± 24.8), spring (450.0 ± 100.0), and autumn (518.2 ± 106.3). No significant differences were noted among winter, spring, and autumn (Figure 3A). Similarly, the Shannon index was highest in summer (4.81 ± 0.30). It significantly exceeded the values observed in winter (3.96 ± 0.23), spring (3.79 ± 0.46), and autumn (3.98 ± 0.53), and no significant differences were detected among these three seasons (Figure 3B). The Simpson index ranged from 0.917 to 0.969, with the highest mean value recorded in summer and the lowest in spring; however, overall seasonal differences were not statistically significant (Figure 3C). Pielou’s evenness index was highest in summer (0.734 ± 0.040) and lowest in spring (0.622 ± 0.057). Evenness was significantly higher in summer than in spring, but the values in winter (0.665 ± 0.033) and autumn (0.638 ± 0.080) did not differ significantly from those of the other seasons (Figure 3D). Overall, summer communities were characterized by higher ASV richness and Shannon diversity, while the Simpson index showed no significant seasonal variation. Significant differences in Pielou’s evenness were only detected between summer and spring.

### 3.3. Seasonal Variation in Taxonomic Composition

**Figure 4:**
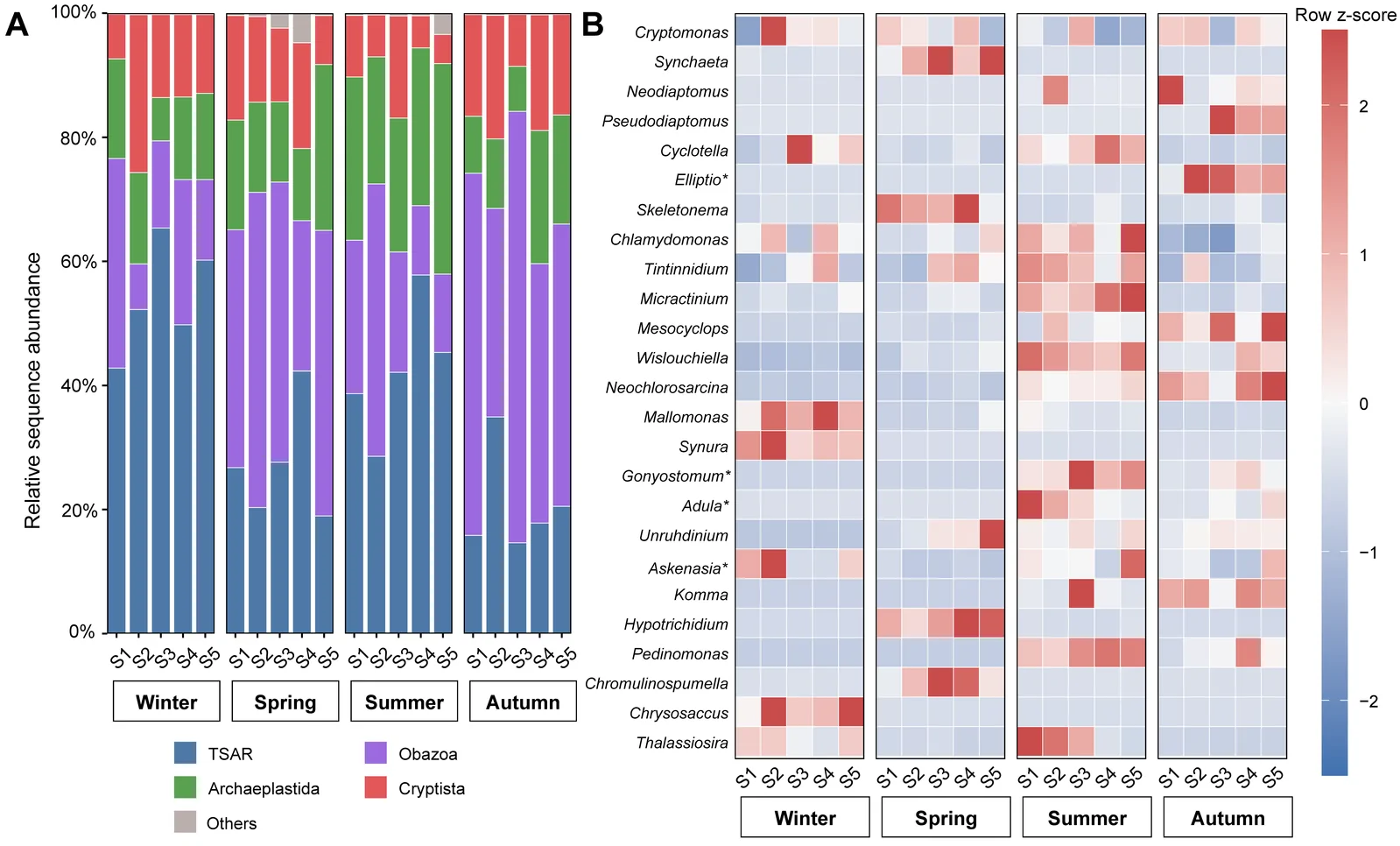
Seasonal variation in supergroup-level taxonomic composition and distribution patterns of dominant genera within aquatic eukaryotic communities. (**A**) Relative sequence abundance of aquatic eukaryotic communities aggregated at the supergroup level. Low-abundance supergroups and sequences lacking reliable supergroup-level taxonomic assignments were pooled into the “others” category. (**B**) Row-wise Z-score heatmap of the top 25 genera ranked by overall mean relative sequence abundance. In panel B, colors represent row-standardized relative sequence abundance values for each genus. Positive and negative values indicate that the relative sequence abundance of a given genus in the corresponding sample is higher or lower than its mean abundance across all samples, respectively. Genus-level analyses included only taxa with annotation confidence ≥ 0.70 and detected in at least 5 of the 20 samples. Genus-level taxonomic classification relied on the PR2 database. Asterisks (*) mark genera for which the representative ASVs exhibited taxonomic discrepancies between PR2 annotations and the top named matches retrieved via NCBIBLASTn (version.2.17.0). Full details are provided in Table S10.

The relative sequence abundances of the major aquatic eukaryotic supergroups varied across the four sampling seasons (Figure 4A). In winter, TSAR had the highest mean relative sequence abundance (54.08% ± 8.86%), followed by Obazoa (18.31% ± 10.39%), Cryptista (14.41% ± 6.70%), and Archaeplastida (13.00% ± 3.52%). In spring, Obazoa accounted for the largest fraction (40.91% ± 10.33%), followed by TSAR (27.20% ± 9.28%) and Archaeplastida (16.71% ± 6.05%). In summer, TSAR and Archaeplastida displayed relatively high mean relative sequence abundances (42.50% ± 10.61% and 25.55% ± 5.30%, respectively), while Obazoa contributed 22.37% ± 13.21%. In autumn, Obazoa reached its peak mean relative sequence abundance (49.77% ± 14.20%), whereas TSAR accounted for 20.74% ± 8.23%, followed by Cryptista (15.92% ± 4.49%) and Archaeplastida (13.34% ± 5.97%). Collectively, TSAR dominated in winter and summer, while Obazoa attained higher sequence proportions in spring and autumn. At the division level, Opisthokonta exhibited the highest relative sequence abundance (32.84%), followed by Chlorophyta (17.14%) and Stramenopiles (17.03%) (Figure S1).

The row-wise Z-score heatmap of the top 25 genera ranked by relative sequence abundance further elucidated seasonal distribution patterns at the genus level (Figure 4B). In winter samples, genera such as *Mallomonas*, *Synura*, *Chrysosaccus*, and *Askenasia* exhibited relatively high sequence abundances. Spring samples were characterized by the relative enrichment of *Hypotrichidium*, *Skeletonema*, *Synchaeta*, and *Chromulinospumella*. During summer, genera including *Micractinium*, *Wislouchiella*, *Pedinomonas*, *Gonyostomum*, *Adula*, *Chlamydomonas*, *Tintinnidium*, and *Cyclotella* demonstrated higher standardized relative sequence abundances (Figure 4B). In autumn, the primarily enriched genera included *Elliptio*, *Neochlorosarcina*, *Mesocyclops, Pseudodiaptomus*, *Komma*, and *Neodiaptomus*. Among these genera, *Cryptomonas* maintained a high relative sequence abundance across all four sampling periods, and its seasonal variation was less pronounced than that of the genera exhibiting distinct period-specific enrichment patterns (Figure 4B). Overall, both the composition of eukaryotic supergroups and the distribution patterns of high-abundance genera indicated substantial taxonomic turnover in aquatic eukaryotic communities across different sampling seasons.

### 3.4. Seasonal Differentiation of Community Composition and Indicator Genera

**Figure 5:**
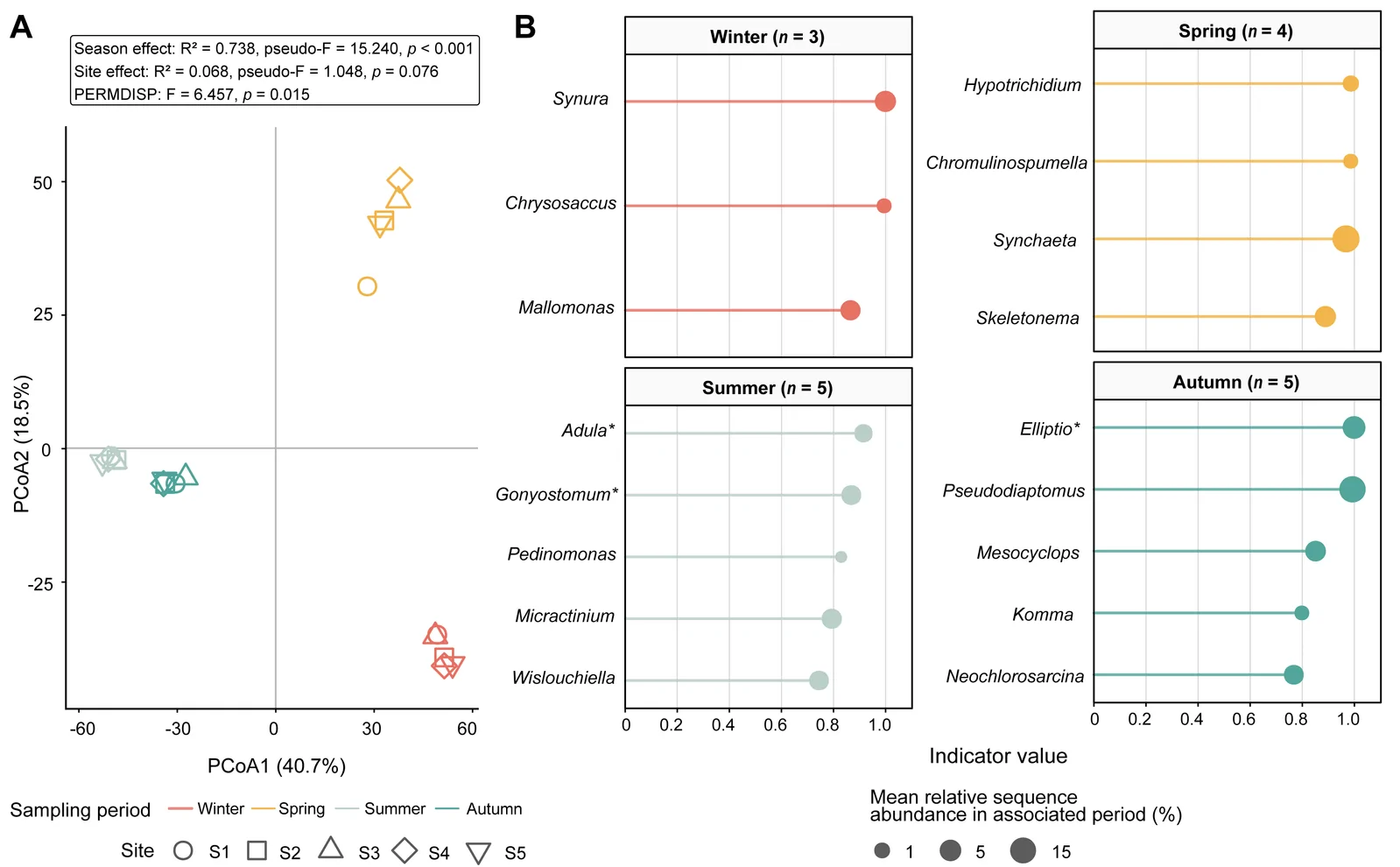
Seasonal differentiation of aquatic eukaryotic communities and seasonal indicator genera. (**A**) Aitchison PCoA based on the CLR-transformed ASV composition. Different colors represent sampling periods, and different point shapes represent sampling sites. (**B**) IndVal analysis of seasonal indicator genera. Only genera with adjusted *p* < 0.05 after Benjamini–Hochberg correction and an overall mean relative sequence abundance ≥ 0.20% are shown. The *x*-axis represents IndVal values, and point size indicates the mean relative sequence abundance of each genus during its associated sampling period. Indicator genera were identified using the original PR2 taxonomic assignments. Asterisks (*) denote genera with genus-level taxonomic discrepancies revealed by NCBI BLASTn analysis of representative ASVs (Table S10).

Aitchison PCoA revealed a distinct separation of aquatic eukaryotic communities across the four seasonal surveys. The first two principal coordinates (PCoA1 and PCoA2) accounted for 40.7% and 18.5% of the variation in community composition, respectively (Figure 5A). Winter and spring samples clustered mainly along the positive side of PCoA1, whereas summer and autumn samples were distributed primarily on the negative side of this axis. Notably, spring samples shifted toward the positive direction of PCoA2, whereas winter samples were largely distributed along the negative direction of PCoA2 (Figure 5A). After controlling for the effect of sampling site, the sampling period accounted for 73.8% of the variation in community composition and demonstrated a statistically significant effect. In contrast, when controlling for the sampling period, the sampling site explained only 6.8% of the community variation and did not achieve statistical significance (Figure 5A). The PERMDISP analysis revealed significant differences in within-group dispersion among sampling periods, indicating that seasonal variations in community composition were characterized not only by separation in multivariate space among groups but also by changes in within-group heterogeneity (Figure 5A).

Under the criteria of an adjusted *p* < 0.05 after Benjamini–Hochberg correction and an overall mean relative sequence abundance ≥ 0.20%, 17 candidate seasonal indicator genera were identified (Figure 5B). The genera associated with winter included *Synura*, *Chrysosaccus*, and *Mallomonas*, which exhibited IndVal values of 0.999, 0.994, and 0.865, respectively. Among these, the mean relative sequence abundances of *Synura* and *Mallomonas* during winter were 4.55% and 3.48% (Figure 5B), respectively. The spring-associated genera consisted of *Hypotrichidium*, *Chromulinospumella*, *Synchaeta*, and *Skeletonema*, with IndVal values ranging from 0.889 to 0.987. Notably, *Synchaeta* demonstrated the highest mean relative sequence abundance in spring at 19.45% (Figure 5B), making it the most abundant spring-associated genus. The summer-associated genera were *Adula*, *Gonyostomum*, *Pedinomonas*, *Micractinium*, and *Wislouchiella*, with IndVal values ranging from 0.744 to 0.915. The mean relative sequence abundances of *Micractinium*, *Gonyostomum*, and *Wislouchiella* in summer were 3.44%, 3.04%, and 2.89% (Figure 5B), respectively. Autumn-associated genera consisted of *Elliptio*, *Pseudodiaptomus*, *Mesocyclops*, *Komma*, and *Neochlorosarcina*, with IndVal values ranging from 0.768 to 0.999. Among these, *Pseudodiaptomus* and *Elliptio* exhibited mean relative sequence abundances of 16.59% and 6.94% in autumn, respectively. These results indicate that different sampling seasons were characterized not only by distinct overall community structures but also by unique sets of indicator genera exhibiting strong season-specific associations.

### 3.5. Seasonal ASV Sharing Patterns and β-Diversity Partitioning

**Figure 6:**
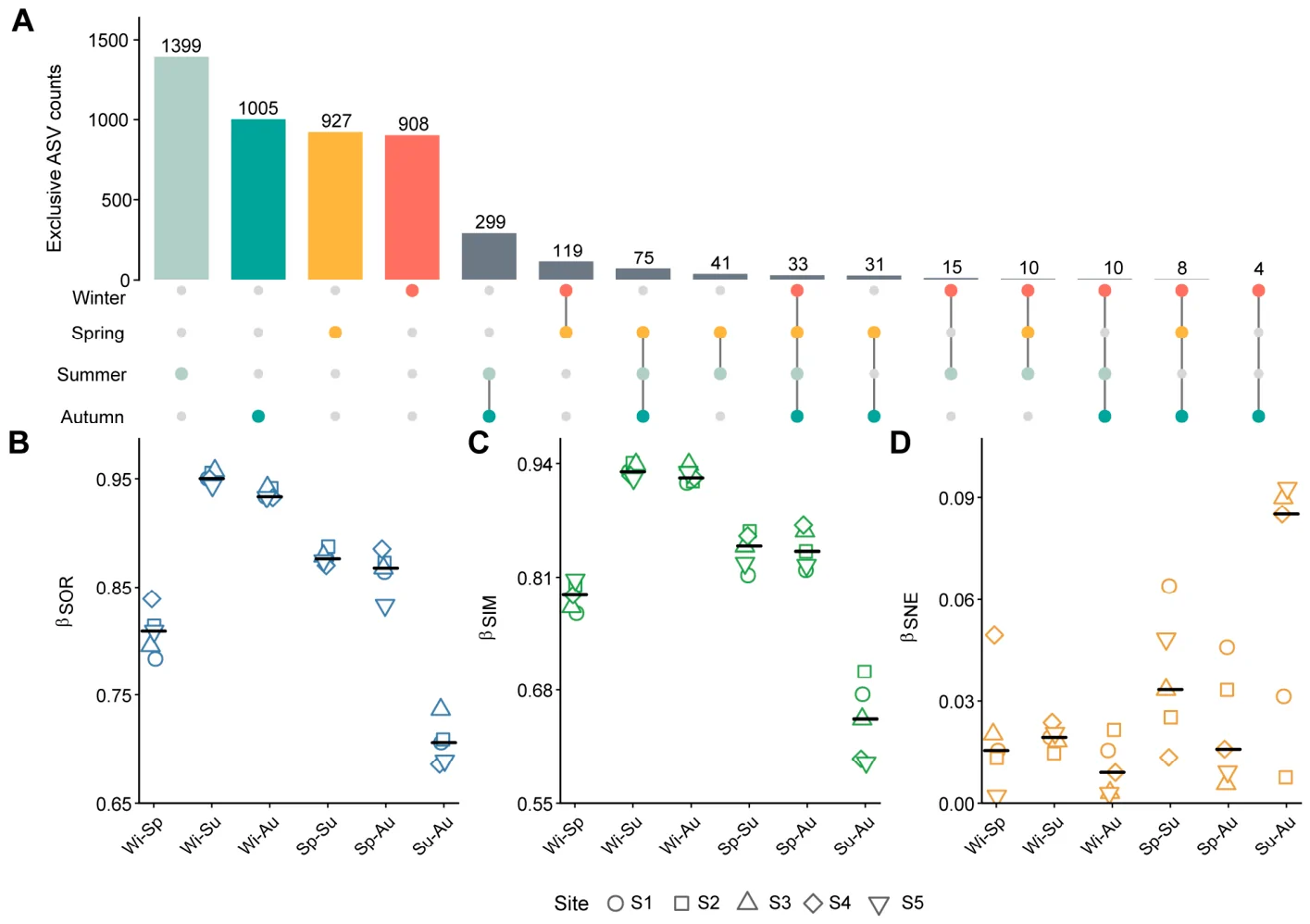
ASV sharing patterns and beta diversity partitioning of aquatic eukaryotic communities across four sampling seasons. (**A**) UpSet diagram illustrating exclusive ASV intersections among sampling seasons. Bar heights correspond to the number of ASVs unique to each seasonal combination, and linked filled dots denote the seasons included in each intersection. (**B**) Overall Sørensen dissimilarity (βSOR); (**C**) Simpson turnover component (βSIM); and (**D**) nestedness-resultant component (βSNE). In panels (**B**–**D**), each point corresponds to a pairwise comparison between two seasons at the same sampling site (*n* = 5). Distinct shapes denote different sampling sites, and black horizontal lines mark median values. Wi, Sp, Su, and Au indicate winter, spring, summer, and autumn, respectively.

The ASV composition showed pronounced seasonal differentiation across the four sampling seasons. The numbers of season-specific ASVs in winter, spring, summer, and autumn were 908, 927, 1399, and 1005, respectively, while only 33 ASVs were shared in all four seasons. The quantity of unique ASVs was substantially greater than that of ASVs shared among different seasons. Among the pairwise seasonal combinations, summer and autumn exhibited the highest number of shared ASVs (299), followed by winter and spring (119 for both). The numbers of shared ASVs among the other seasonal combinations were relatively low (Figure 6A). These findings suggest that the proportion of ASVs detected across all four sampling seasons in the Qianmudang Reservoir was relatively low, with many ASVs displaying seasonal appearance and disappearance patterns.

Pairwise comparisons among sampling periods at the same sites revealed generally high βSOR values, with median values ranging from 0.706 to 0.950 across seasonal comparisons (Figure 6B). Among all seasonal pairs, the winter–summer comparison exhibited the highest βSOR (median = 0.950), followed by winter–autumn (median = 0.933), while summer–autumn showed the lowest value (median = 0.706). βSIM demonstrated a similar pattern to βSOR, with median values ranging from 0.647 to 0.931 across seasonal comparisons. The highest βSIM was observed between winter and summer (median = 0.931), while the lowest value occurred between summer and autumn (median = 0.647) (Figure 6C). In contrast, βSNE consistently exhibited low values, with median values ranging from 0.009 to 0.085. βSNE was relatively higher between summer and autumn, whereas all other seasonal comparisons exhibited values < 0.04 (Figure 6D). Overall, the high community dissimilarity among sampling periods was primarily driven by ASV turnover, while the contribution of nestedness-resultant dissimilarity in community richness was relatively limited.

### 3.6. ASV Occupancy Patterns and Occupancy-Abundance Relationships

**Figure 7:**
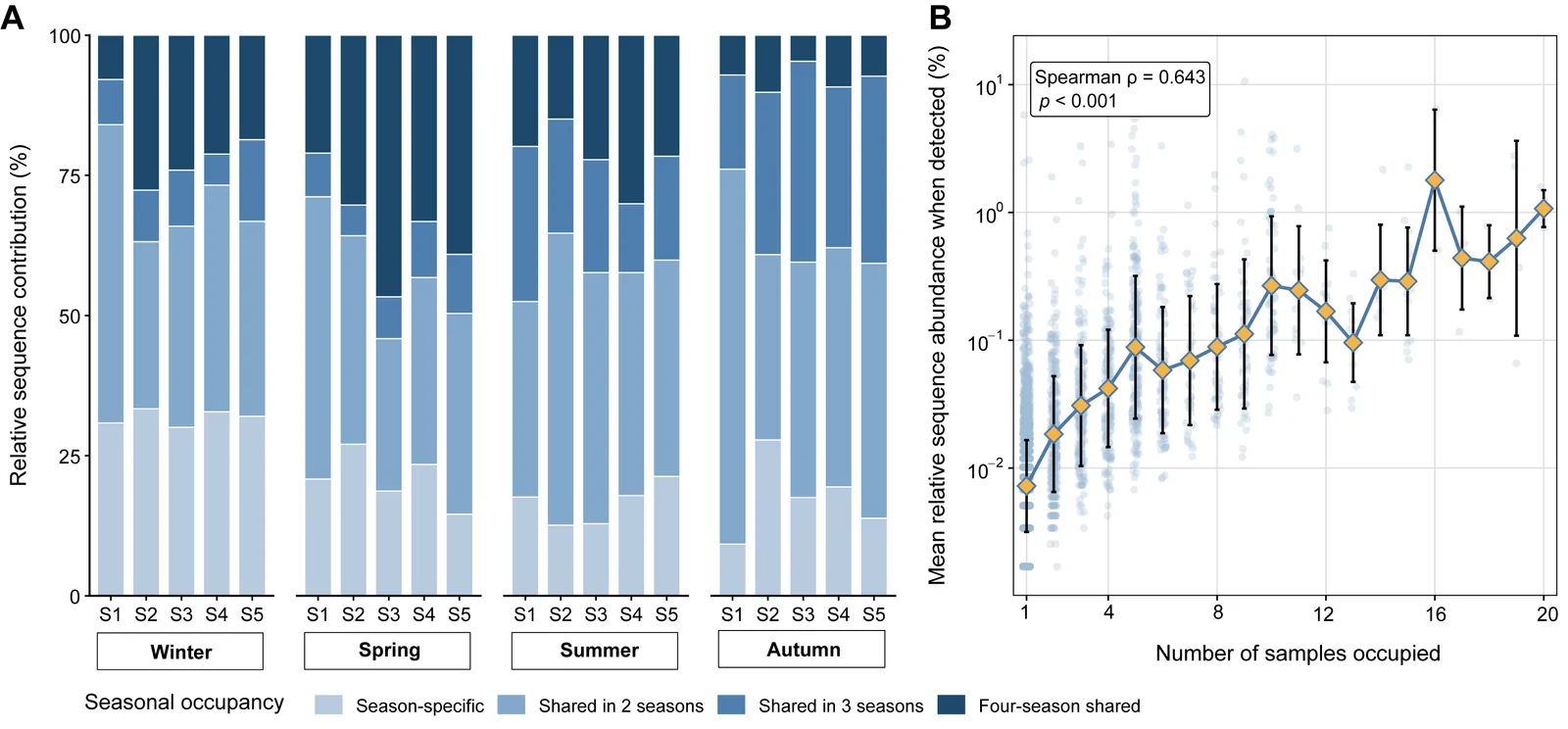
Seasonal occupancy patterns and occupancy-abundance relationships of aquatic eukaryotic ASVs. (**A**) Relative sequence contributions of different seasonal occupancy categories across sampling periods and sites. Single-period, two-period shared, three-period shared, and four-period shared ASVs represent ASVs detected in 1, 2, 3, and 4 sampling periods, respectively. Each bar represents an individual sample, and the contributions of all occupancy categories were normalized to 100%. (**B**) Relationship between ASV sample occupancy and the mean relative sequence abundance when detected. Light blue points represent individual ASVs. Yellow diamonds represent geometric means obtained by back-transforming the means of log10-transformed abundances at each occupancy level. Black error bars indicate the back-transformed range of the log_10_-transformed mean ± 1 standard deviation. The blue line represents the overall trend of geometric means. Correlations were assessed using Spearman’s rank correlation analysis.

Detected ASVs were categorized into four groups: single-season ASVs, two-season shared ASVs, three-season shared ASVs, and four-season shared ASVs. The contributions of different occupancy categories to the relative sequence abundance of each sample exhibited distinct differences; however, all fixed sampling sites demonstrated similar overall compositional patterns (Figure 7A). Among the 20 samples analyzed, two-season shared ASVs showed the highest mean sequence contribution, with an average of 40.90%. The mean contributions of single-season-specific ASVs, four-season shared ASVs, and three-season shared ASVs were 21.69%, 20.85%, and 16.56% (Figure 7A), respectively.

The contribution structure of different occupancy categories varied across sampling periods. In winter, single-period ASVs contributed an average of 31.82%, which was higher than their contributions in spring, summer, and autumn. In spring, four-period shared ASVs exhibited the highest mean contribution among all sampling periods, reaching 34.07% (Figure 7A). Summer communities were primarily characterized by two-period shared ASVs, which had an average contribution of 42.01%. In autumn, the mean contributions of two-period shared and three-period shared ASVs increased to 46.02% and 28.72%, respectively, while the contribution of four-period shared ASVs decreased to 7.70% (Figure 7A). These results, in conjunction with the previously mentioned ASV intersection patterns, indicate that although ASVs detected in only one sampling period accounted for the majority of ASV richness, their sequence contributions did not increase proportionally. In contrast, ASVs shared across multiple sampling periods, despite being fewer in number, contributed a relatively large proportion of total sequences. This finding suggests that the numerical composition of ASVs and their sequence contribution structure are not equivalent and that the contribution to community sequence composition of different occupancy categories cannot be inferred solely from ASV counts.

The number of samples occupied by each ASV exhibited a significant positive correlation with its mean relative sequence abundance when detected (Spearman’s ρ = 0.643, *p* < 0.001; Figure 7B). As ASV sample occupancy increased from 1 to 20 samples, the mean relative sequence abundance of detected ASVs also generally exhibited an increasing trend, although considerable variation persisted across different occupancy levels. ASVs detected in a limited number of samples were predominantly concentrated within lower abundance ranges, whereas ASVs occupying a greater number of samples typically demonstrated higher mean relative sequence abundances. These findings further suggest that high-occupancy ASVs, despite being relatively few in number, accounted for a substantial proportion of community sequences. Conversely, low-occupancy ASVs constituted a numerically large yet individually low contributing group (Figure 7B). This distinction establishes a basis for further evaluation of the contributions of low- and high-occupancy ASVs to community sequence composition.

### 3.7. Associations Between Environmental Variables and Community Composition

**Figure 8:**
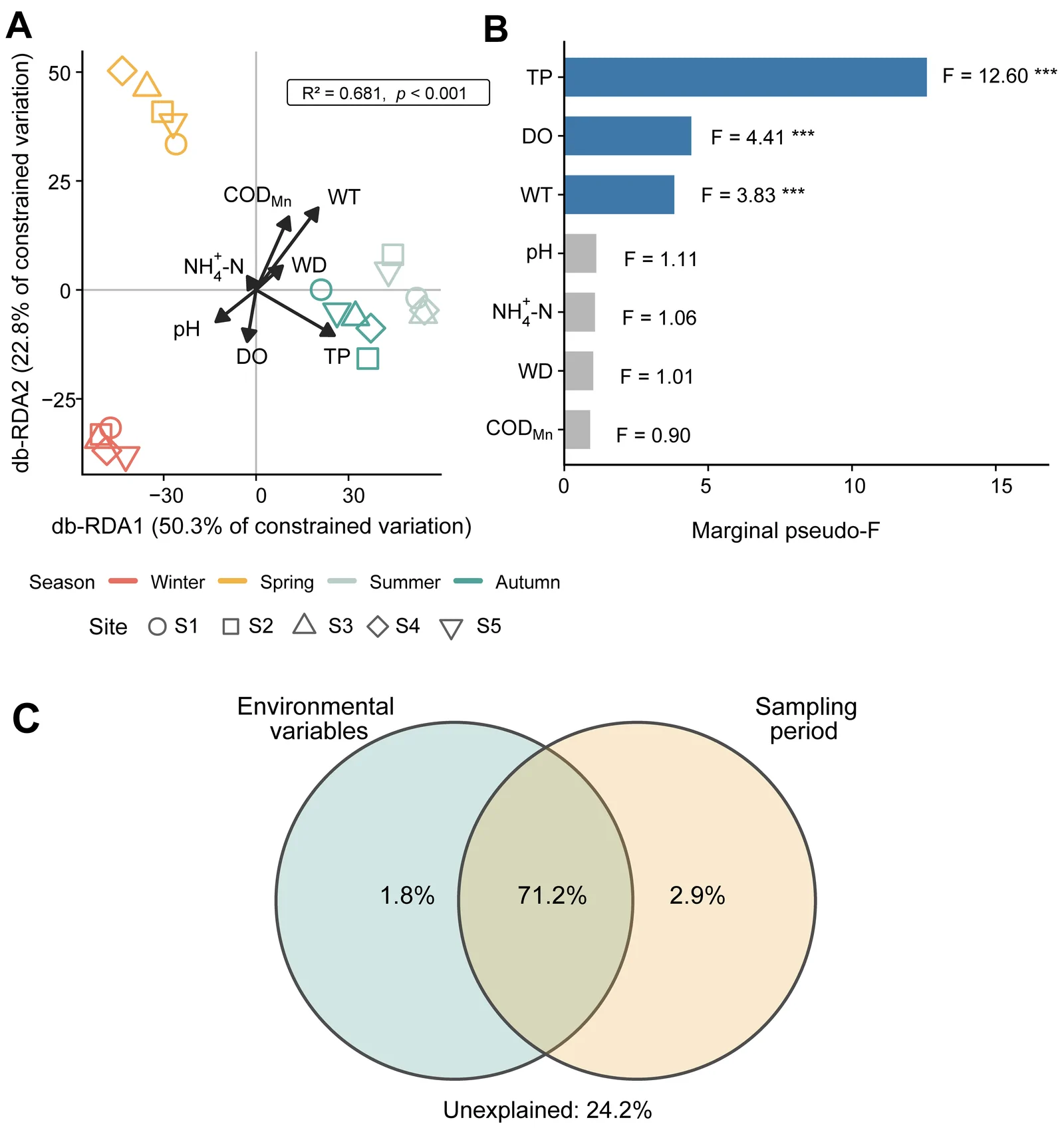
Relationships between aquatic eukaryotic community composition and environmental variables. (**A**) Partial db-RDA ordination plot with sampling site included as a conditioning variable. Different colors represent seasons, and different shapes represent fixed sampling sites. The direction of arrows indicates the increasing direction of environmental variables, and arrow length reflects their correlation strength with the ordination axes. (**B**) Marginal association strength of seven VIF-selected environmental variables with community composition, represented by marginal pseudo-*F* values. Blue bars indicate environmental variables that remained significant after Benjamini–Hochberg correction, whereas gray bars indicate variables that did not reach statistical significance. *** indicates adjusted *p* < 0.001 after Benjamini–Hochberg correction. (**C**) Variation partitioning analysis (VPA) disentangling the effects of sampling period and environmental variables after accounting for sampling site. Values indicate the unique and shared fractions of site-adjusted community variation, quantified by adjusted R^2^. The unexplained fraction is presented outside the circles.

After accounting for differences among sampling sites, partial db-RDA revealed a distinct separation of aquatic eukaryotic communities across different seasons in the ordination space (Figure 8A). Winter and spring samples were predominantly distributed along the negative axis of db-RDA1, with spring samples located on the positive axis of db-RDA2 and winter samples positioned on the negative axis. In contrast, summer and autumn samples were primarily distributed along the positive axis of db-RDA1 and exhibited further separation along the db-RDA2 axis. The first two constrained axes, db-RDA1 and db-RDA2, explained 50.3% and 22.8% of the constrained variation (Figure 8A), respectively. The overall environmental model was significant (adjusted R^2^ = 0.681, pseudo-F = 6.790, *p* < 0.001), indicating that the retained environmental variables were significantly associated with variations in community composition.

Environmental vectors indicated that various environmental variables were linked to distinct patterns of community variation. TP was primarily oriented towards the positive direction of db-RDA1 and the negative direction of db-RDA2, while WT and COD_Mn_ were predominantly aligned with the positive directions of both axes (Figure 8A). DO and pH were mainly associated with the negative direction of db-RDA2. Marginal tests further demonstrated that TP exhibited the strongest association with community composition, evidenced by a marginal pseudo-F value of 12.60. DO and WT followed with the next strongest associations, reflected by marginal pseudo-F values of 4.41 and 3.83, respectively. Notably, all three variables remained significant after the Benjamini–Hochberg correction (Figure 8B). In contrast, the marginal effects of pH, NH_4_^+^−N, WD, and COD_Mn_ did not reach statistical significance (Figure 8B). Overall, TP showed the strongest marginal association with community composition among the seven VIF-screened environmental predictors, followed by DO and WT.

Further variation partitioning analysis (VPA) showed a large overlapping fraction of explained variation between sampling period and environmental variables after conditioning on sampling site (Figure 8C). This shared component accounted for 71.2% of the site-adjusted community variation, whereas environmental variables and sampling period independently explained only 1.8% and 2.9%, respectively. The remaining 24.2% of variation remained unexplained. Moreover, when both sampling period and sampling site were partialled out simultaneously, the global environmental model was not statistically significant (pseudo-F = 1.130, *p* = 0.1299). Collectively, these findings suggest that the environmental associations detected by the site-conditioned partial db-RDA largely overlapped with seasonal variation, and that environmental variables conferred only a modest independent explanatory effect on community composition.

## 4. Discussion

### 4.1. Seasonal Environmental Variation and Differentiation of Aquatic Eukaryotic Communities

During the four-season surveys conducted in Qianmudang Reservoir in 2025, the physicochemical parameters exhibited pronounced seasonal variations. Environmental characteristics, including WT, nutrient availability, DO, and transparency, varied significantly among sampling seasons (Figure 2), providing distinct environmental contexts for seasonal changes in aquatic eukaryotic communities. Under these fluctuating environmental conditions, community α-diversity, major taxonomic composition, and genus-level composition demonstrated varying degrees of temporal variation (Figure 3 and Figure 4), which were accompanied by significant differentiation in the overall community structure. Similar patterns have been reported in previous studies conducted in Zhaoshandu Reservoir and southern Lake Taihu by Zhao et al. (2026) and Zuo et al. (2025) [18,21].

The α-diversity indices of aquatic eukaryotic communities in Qianmudang Reservoir exhibited distinct seasonal responses (Figure 3). ASV richness and the Shannon index were significantly higher in summer compared to the other three seasons (Figure 3A,B), whereas Pielou’s evenness showed a significant increase in summer relative to spring (Figure 3D). In contrast, significant seasonal differences in the Simpson index were not detected (Figure 3C). Thus, the unique diversity characteristics observed in summer were primarily indicated by an increase in detectable ASV numbers, which was accompanied by elevated Shannon diversity. However, different α-diversity metrics did not display entirely consistent seasonal response patterns. Similar seasonal variation patterns have been documented by Zhang et al. (2023) in the Jinpen Reservoir, where eukaryotic diversity reached its annual peak in summer, with taxa such as *Rimostrombidium* significantly contributing to the relative sequence composition during this period [42]. A four-season investigation in the Chaohu Lake basin also revealed that aquatic eukaryotic communities exhibited the highest α-diversity in summer [9], which aligns with the increased richness and Shannon diversity observed in Qianmudang Reservoir during this season (Figure 3A,B). However, summer does not universally represent the period of maximum eukaryotic diversity across all freshwater ecosystems. Cai et al. (2025) reported that the highest α-diversity of aquatic eukaryotic communities in an urban river system of the Xiangjiang River primarily occurred in spring and winter, while diversity was comparatively lower in summer [43]. These discrepancies suggest that seasonal peaks in freshwater eukaryotic diversity are not universal across aquatic systems but rather exhibit ecosystem-specific response patterns. This further underscores the necessity for comprehensive investigations across various ecologically significant water bodies.

In addition to α-diversity, the taxonomic composition of aquatic eukaryotic communities in Qianmudang Reservoir exhibited pronounced seasonal shifts (Figure 4). At the supergroup level, TSAR dominated in winter and summer with the highest relative sequence abundance, while Obazoa accounted for the largest proportion in spring and autumn (Figure 4A). Genus-level composition and IndVal analyses further indicated that distinct high-abundance genera and season-associated indicator genera characterized different sampling seasons (Figure 4B and Figure 5B). Similar seasonal shifts in eukaryotic groups have been reported in the Han River, South Korea. Choi et al. (2022) observed that diatoms in the order Fragilariales exhibited high sequence abundance in winter [44], followed by a decline in spring that was accompanied by an increase in Thalassiosirales. They also reported that Aulacoseirales increased during summer, reaching higher levels in autumn. Meanwhile, the green algal order Sphaeropleales demonstrated relatively high abundance primarily in spring and summer, while Arthropoda were more frequently observed in summer and autumn, with Calanoida and Ploima exhibiting higher relative abundances in summer and autumn, respectively [44]. Yang et al. (2024) also reported that Chlorophyta exhibited higher relative sequence abundances during winter and spring in the Jihongtan Reservoir but experienced a substantial decline in summer and autumn [45]. In contrast, Arthropoda, particularly its dominant genus *Paracyclopina*, showed the lowest relative abundance in winter and a marked increase in summer [45]. Although those investigations profiled aquatic eukaryotic communities at distinct taxonomic resolutions, they consistently reported seasonal shifts in community composition, with divergent seasonal patterns across taxonomic groups.

Moreover, the temporal variations in α-diversity and taxonomic composition observed in Qianmudang Reservoir were consistent with significant differentiation in overall community structure (Figure 4 and Figure 5A). After controlling for the effect of sampling site, the sampling season accounted for 73.8% of the variation in community composition in the PERMANOVA analysis. In contrast, after controlling for the effect of sampling period, the sampling site explained only 6.8% of the community variation (Figure 5A), which did not reach statistical significance. The PERMDISP analysis further revealed significant differences in within-group dispersion among sampling seasons (Figure 5A). PERMANOVA indicated pronounced seasonal differentiation in community composition. However, the significant PERMDISP result suggests that this pattern reflected both differences in group centroids and seasonal changes in within-group dispersion. Freshwater eukaryotic communities generally exhibit both temporal and spatial heterogeneity; however, the relative importance of these dimensions varies among aquatic systems and across different spatial and temporal scales. Seasonal surveys conducted in Qingcaosha Reservoir and Dongqian Lake also revealed pronounced seasonal differentiation, with seasonal variation exerting a stronger influence on community structure than spatial differences among sampling locations [5,46]. These findings suggest that temporal environmental variation can surpass the spatial heterogeneity caused by sampling locations within a water body. Similarly, Li et al. (2022) investigated the middle route of the South-to-North Water Diversion Project and reported that season explained 41% of the variation in community composition based on Adonis analysis [47]. Further variation partitioning indicated that the independent contribution of season was 13%, exceeding that of geographic distance (8%). Moreover, the shared contribution between environmental factors and season was 6%, which was also higher than the shared contribution between environmental factors and spatial distance (1%) [47]. Together, these studies indicate that temporal variation in aquatic eukaryotic communities can exceed spatial differences within the investigated systems.

In the present study, the results obtained for Qianmudang Reservoir revealed that differences among sampling seasons were more pronounced than differences among sampling sites within the sampling design (Figure 5A). This pattern may be attributed to the limited spatial extent of the study area and the high connectivity and water exchange within Qianmudang Reservoir. Additionally, Li et al. (2022) suggested that under dynamic water transport conditions, stable water flow may facilitate the dispersal of eukaryotic organisms across spatial barriers, thereby reducing the effects of geographic dispersal limitation [47]. However, because flow velocity and water exchange processes were not directly measured in the present study, this interpretation necessitates further validation.

When interpreting these seasonal community patterns, potential methodological caveats associated with 18S rRNA V4 amplicon sequencing should be acknowledged, because amplification efficiency, rDNA copy number, and taxonomic resolution differ across eukaryotic lineages [48,49]. In the present study, the filtration procedure omitted prefiltration, which permitted the recovery of metazoan sequences. The complementary analysis at the division level further delineated dominant eukaryotic groups and their seasonal shifts in relative sequence abundance (Figure S1), offering a broader taxonomic view of community composition. BLASTn validation of representative ASVs from the top 25 genera (including the 17 indicator genera) indicated that most representative sequences yielded genus-level matches congruent with PR2 taxonomic annotations, though several mismatches and ambiguous assignments were detected (Table S10). These observations highlight that abundant genera and seasonal indicator taxa should be interpreted with reference to their PR2 classifications, relative sequence contributions, and seasonal associations, while acknowledging inherent uncertainties in genus-level taxonomic identification. Notably, after removing Metazoa-assigned ASVs, ASV richness and Shannon diversity still peaked in summer; PCoA and PERMANOVA recovered comparable seasonal differentiation, and ASV turnover remained the dominant component of β-diversity (Figures S2–S4; Tables S3–S9). Such consistency reinforces the robustness of our core conclusions concerning seasonal community turnover and ASV dynamics in Qianmudang Reservoir.

### 4.2. Seasonal ASV Turnover and Occupancy Patterns

At the ASV level, the seasonal restructuring of aquatic eukaryotic communities in Qianmudang Reservoir was primarily characterized by ASV turnover (Figure 6), while the contribution of nestedness-resultant dissimilarity in community richness was relatively limited. Only 33 ASVs were shared across all four sampling seasons, whereas 908, 927, 1399, and 1005 season-specific ASVs were detected in winter, spring, summer, and autumn (Figure 6A), respectively. In pairwise comparisons between different periods at the same sampling sites, the median values of the turnover component (βSIM) ranged from 0.647 to 0.931, while those of the nestedness component (βSNE) ranged only from 0.009 to 0.085 (Figure 6B–D). This pattern persisted following the removal of Metazoa-assigned ASVs (Figure S4). The greatest ASV turnover was detected between winter and summer, while the lowest turnover occurred between summer and autumn. Seasonal community changes in Qianmudang Reservoir were not primarily driven by directional increases or decreases in ASV richness; rather, they involved extensive temporal replacement of ASV members. Similar patterns have been reported in other studies of aquatic eukaryotic communities [50,51]. For instance, Xue et al. (2018) found pronounced temporal turnover in both abundant and rare eukaryotic communities, with stronger turnover observed in rare taxa in Xidong Reservoir [50]. Further β-diversity partitioning also indicated that member turnover contributed more significantly than richness differences to community variation in both community types [50]. Wang et al. (2025) also found that the β-diversity of rare eukaryotic communities in surface waters of Hengshan Reservoir was primarily driven by turnover, whereas the contribution of nestedness increased in deep-water communities [51]. Collectively, these findings indicate that ASV turnover may represent an important pattern of aquatic eukaryotic community restructuring. The consistently higher βSIM than βSNE observed in Qianmudang Reservoir further demonstrates that the high community dissimilarity among the four seasonal surveys was primarily attributable to the replacement of ASV identities (Figure 6C,D), while the contribution of nestedness associated with richness differences was relatively limited.

The differences in the number and relative sequence contributions of ASVs with varying seasonal occurrence ranges further elucidate the internal structure underlying seasonal community restructuring. In Qianmudang Reservoir, the number of ASVs detected in only one sampling season were higher than those shared by different seasons, but their average sequence contribution across all samples was only 21.69% (Figure 7A). In contrast, ASVs shared across two seasons contributed an average of 40.90%, while those shared across four seasons accounted for 20.85% of the average sequence contribution (Figure 7A). Furthermore, ASV sample occupancy across the 20 samples was significantly and positively correlated with the mean relative sequence abundance when detected (ρ = 0.643, *p* < 0.001) (Figure 7B). ASVs detected in only a small number of samples were primarily concentrated within lower abundance ranges, whereas ASVs occurring in more samples generally exhibited higher mean relative sequence abundances (Figure 7B). This occupancy-abundance relationship should be interpreted descriptively because low-abundance ASVs are inherently less likely to be detected consistently across samples.

The aforementioned pattern has also been reported in eukaryotic communities of other freshwater bodies [52,53]. For example, Li et al. (2025) conducted a 1-year 18S rDNA survey in Lake Weishan and classified eukaryotic taxa into habitat-generalist and habitat-specialist groups [52]. The specialist group contained 425 ASVs, while the generalist group included only 189 ASVs, indicating that eukaryotic members with narrower ecological distributions can represent a substantial proportion of community membership [52]. Although the specialist classification employed in that study does not precisely align with the seasonal and sample occupancy definitions utilized herein, both studies illustrate that narrowly distributed members can be more numerous in terms of taxon richness. Xue et al. (2023) identified a significant positive correlation between ASV occurrence frequency and mean relative abundance in Tingxi Reservoir [53]. The core taxa, characterized by high occurrence frequencies, were fewer in number but contributed substantially to overall abundance, while satellite taxa, with low occurrence frequencies, primarily consisted of numerous low-abundance members [53]. Therefore, when considering both seasonal occurrence range and sample occupancy, ASVs with restricted distributions in Qianmudang Reservoir were more numerous, whereas high-occupancy ASVs generally exhibited higher mean relative sequence abundances when detected (Figure 7). This finding suggests that ASV numbers alone cannot accurately reflect the contributions of different members to community sequence composition. It is important to note that the contributions discussed herein refer to relative sequence contributions obtained from 18S rRNA gene amplicon sequencing and should not be directly interpreted as equivalent to cell abundance, biomass, or ecological functional contribution.

### 4.3. Environmental Associations with Eukaryotic Community Composition

Partial db-RDA analysis further demonstrated that community composition in Qianmudang Reservoir was significantly associated with the environmental parameters (Figure 8). After controlling for differences among sampling sites, TP exhibited the strongest marginal association with community composition, followed by DO and WT (Figure 8B). All three variables remained significant after correction for multiple testing (Figure 8B). Further variation partitioning analysis revealed a considerable shared fraction between environmental variables and sampling period (Figure 8C). These findings indicate that the environmental associations detected via partial db-RDA were tightly intertwined with seasonal variation.

The close relationship between phosphorus availability and eukaryotic community composition has also been documented in other aquatic systems. For instance, Zhang et al. (2023) found that eukaryotic community composition shifted with increasing phosphorus availability in a riverine-coastal continuum [54]. They also found a notable increase in the relative abundance of Chlorophyta, which represents an important component of this transition [54]. Chen et al. (2025) also revealed that environmental variables, including TP, were closely linked to the spatial and temporal distribution of eukaryotic plankton communities [55]. These studies collectively suggest that phosphorus availability can shape community composition and modulate the relative sequence contributions of distinct eukaryotic groups in freshwater ecosystems.

Additionally, Zhao et al. (2026) reported that WT was closely associated with the eukaryotic community structure and temporal variation in Zhaoshandu Reservoir [21]. Cai et al. (2025) also reported a significant association between WT and eukaryotic community composition in Xiangjiang River [43]. Zheng et al. (2020) further demonstrated that multiple environmental variables, including WT and DO, were significantly associated with the composition of planktonic eukaryotic communities in Danjiangkou Reservoir [56]. These results suggest that TP, WT, and DO can be interpreted together to explore the temporal variations in reservoir eukaryotic communities. However, it is important to note that environmental variables, such as TP, WT and DO, displayed strong seasonal synchrony. This study only encompassed one annual cycle, with a single representative survey conducted in each season. Consequently, the observed seasonal differences may reflect effects specific to individual sampling dates, and their inter-annual consistency should be validated through long-term monitoring. Furthermore, partial db-RDA primarily reveals statistical associations between environmental parameters and community composition, but it cannot ascertain the independent causal effects of individual environmental variables. Further investigations across various drinking water source systems are essential to enhance our understanding of the mechanisms driving temporal variation in aquatic eukaryotic community structures.

## 5. Conclusions

Using 18S rRNA gene amplicon sequencing, we investigated seasonal variation, ASV restructuring patterns, and their associations with environmental variables in aquatic eukaryotic communities of Qianmudang Reservoir. The main conclusions are as follows: (1) The aquatic eukaryotic community composition in Qianmudang Reservoir was associated with TP, DO and WT, with TP exhibiting the strongest marginal effect. Meanwhile, a large proportion of the explained variation was shared between environmental variables and sampling period. (2) Aquatic eukaryotic communities in this reservoir showed pronounced seasonal variation, with the highest ASV richness and Shannon diversity observed in summer. (3) Seasonal restructuring of aquatic eukaryotic communities was primarily characterized by ASV turnover rather than nestedness. Single season-specific ASVs accounted for a large proportion of ASV richness, whereas ASVs with higher sample occupancy generally exhibited higher mean relative sequence abundances when detected.

## Data Availability

Data underlying this article had been deposited to the GenBank Nucleotide Database and can be accessed on 31 October 2026 via https://www.ncbi.nlm.nih.gov/search/all/?term=PRJNA1550032.

## Supplementary Materials

The following supporting information can be downloaded at: https://www.mdpi.com/article/10.3390/biology15212036/s1.
