# Supplementary material for: Seasonal Dynamics of Aquatic Eukaryotic Communities in a Yangtze Delta Shallow Drinking-Water Reservoir Revealed by 18S rRNA Gene Metabarcoding
Source: Biology (Basel). 2026 Nov 8;15(21):2036. doi: 10.3390/biology15212036 (PMC13761875; doi:10.3390/biology15212036)
Supplement: Supplementary file 1 [file biology-15-02036-s001.zip › Supplementary_Materials.pdf]

Supplementary Materials

Statistical comparisons among four sampling periods (n = 5 sampling sites per period). The results below provide the overall test statistic and degrees of freedom, both raw and Benjamini–Hochberg (BH)-adjusted omnibus *P*-values, as well as the adjusted *P*-values for all six seasonal pairwise comparisons.

Physicochemical parameters (Section 3.1; Figure 2)

Table S1A. Seasonal values and omnibus tests for 12 physicochemical variables.

Variable	Winter	Spring	Summer	Autumn	Test	Test statistic	df	Raw *P*	BH-adj. *P*
WT (°C)	7.60 ± 0.42	23.40 ± 0.74	28.00 ± 0.70	20.76 ± 0.36	ANOVA	F = 1141.143	3, 16	<0.0001	<0.0001
SD (cm)	66.80 ± 8.70	37.80 ± 2.28	25.80 ± 2.59	24.80 ± 1.48	Kruskal–Wallis	H = 16.228	3	0.0010	0.0017
WD (m)	2.96 ± 0.51	3.12 ± 0.26	3.30 ± 0.19	3.06 ± 0.23	ANOVA	F = 0.976	3, 16	0.4285	0.4285
DO (mg/L)	10.80 ± 0.65	7.82 ± 0.45	10.92 ± 0.54	6.22 ± 0.18	ANOVA	F = 112.652	3, 16	<0.0001	<0.0001
Chl-*a* (μg/L)	23.89 ± 5.97	25.78 ± 2.82	18.88 ± 2.62	6.10 ± 1.60	ANOVA	F = 29.595	3, 16	<0.0001	<0.0001
COD_{Mn} (mg/L)	4.00 ± 0.12	4.92 ± 0.27	5.12 ± 0.86	4.34 ± 0.17	Kruskal–Wallis	H = 10.503	3	0.0147	0.0161
TN (mg/L)	4.08 ± 0.25	2.85 ± 0.14	1.73 ± 0.23	2.44 ± 0.25	ANOVA	F = 98.376	3, 16	<0.0001	<0.0001
NO_3^--N (mg/L)	3.00 ± 0.15	2.08 ± 0.10	1.05 ± 0.17	1.52 ± 0.21	ANOVA	F = 134.131	3, 16	<0.0001	<0.0001
NH_4^+-N (mg/L)	0.119 ± 0.110	0.118 ± 0.042	0.154 ± 0.050	0.032 ± 0.004	Kruskal–Wallis	H = 11.489	3	0.0094	0.0112
TP (mg/L)	0.08 ± 0.02	0.06 ± 0.01	0.12 ± 0.01	0.12 ± 0.01	Kruskal–Wallis	H = 15.238	3	0.0016	0.0024
PO_4^{3-}-P (mg/L)	0.016 ± 0.009	0.010 ± 0.000	0.060 ± 0.010	0.034 ± 0.009	ANOVA	F = 38.769	3, 16	<0.0001	<0.0001
pH	7.84 ± 0.15	7.75 ± 0.14	7.56 ± 0.09	7.82 ± 0.08	ANOVA	F = 5.677	3, 16	0.0076	0.0102

Values are mean ± SD (n = 5 per season). ANOVA is reported as F with df = (3,16); Kruskal–Wallis is reported as H with df = 3. The BH-adjusted omnibus *P*-values were calculated across the 12 physicochemical variables as an additional multiplicity correction for this supplementary analysis.

Table S1B. Adjusted *P*-values for post hoc comparisons of physicochemical variables between sampling periods.

Variable	Post hoc	Win–Spr	Win–Sum	Win–Aut	Spr–Sum	Spr–Aut	Sum–Aut
WT (°C)	Tukey HSD	<0.0001	<0.0001	<0.0001	<0.0001	<0.0001	<0.0001
SD (cm)	Dunn-BH	0.2164	0.0039	0.0030	0.0910	0.0641	0.7887
WD (m)	Tukey HSD	0.8612	0.3739	0.9604	0.8149	0.9909	0.6511
DO (mg/L)	Tukey HSD	<0.0001	0.9772	<0.0001	<0.0001	0.0005	<0.0001
Chl-*a* (μg/L)	Tukey HSD	0.8425	0.1730	<0.0001	0.0385	<0.0001	0.0002
COD_{Mn} (mg/L)	Dunn-BH	0.0302	0.0227	0.2378	0.7477	0.2378	0.2155
TN (mg/L)	Tukey HSD	<0.0001	<0.0001	<0.0001	<0.0001	0.0417	0.0006
NO_3^--N (mg/L)	Tukey HSD	<0.0001	<0.0001	<0.0001	<0.0001	0.0003	0.0017
NH_4^+-N (mg/L)	Dunn-BH	0.6683	0.5873	0.0490	0.6683	0.0222	0.0114
TP (mg/L)	Dunn-BH	0.5015	0.0326	0.0326	0.0057	0.0057	1.0000
PO_4^{3-}-P (mg/L)	Tukey HSD	0.6494	<0.0001	0.0133	<0.0001	0.0012	0.0006
pH	Tukey HSD	0.6428	0.0095	0.9933	0.0967	0.7926	0.0162

Tukey HSD adjusted *P*-values are reported following one-way ANOVA; Dunn test *P*-values were BH-adjusted within the six pairwise comparisons for each variable following Kruskal–Wallis. Winter–Spring etc. identify pairs of seasons (not P-values for individual seasons). $P < 0.05$ indicates a significant pairwise difference.

Alpha-diversity indices (Section 3.2; Figure 3)

Four indices were recalculated directly from the rarefied ASV count matrix (20 samples; 58,954 reads per sample): observed ASV richness, Shannon diversity, Simpson diversity, and Pielou evenness. The test-selection procedure follows the supplied Figure 3 script: Shapiro–Wilk testing of ANOVA residuals and median-centered Levene testing, followed by ANOVA/Tukey HSD or Kruskal–Wallis/Dunn-BH as appropriate.

Table S2A. Seasonal values and omnibus tests for four alpha-diversity indices.

Variable	Winter	Spring	Summer	Autumn	Test	Test statistic	df	Raw *P*	BH-adj. *P*
Richness	389.4 ± 24.8	450.0 ± 100.0	698.0 ± 44.9	518.2 ± 106.3	ANOVA	F = 14.899	3, 16	<0.0001	0.0003
Shannon	3.965 ± 0.226	3.794 ± 0.465	4.808 ± 0.302	3.976 ± 0.531	ANOVA	F = 6.490	3, 16	0.0044	0.0089
Simpson	0.953 ± 0.014	0.917 ± 0.039	0.969 ± 0.019	0.921 ± 0.076	Kruskal–Wallis	H = 6.749	3	0.0804	0.0804
Pielou	0.665 ± 0.033	0.622 ± 0.057	0.734 ± 0.040	0.638 ± 0.080	ANOVA	F = 4.000	3, 16	0.0266	0.0355

Values are mean ± SD (n = 5 per season). For ANOVA, F has df = (3, 16); for Kruskal–Wallis, H has df = 3. BH-adjusted omnibus *P*-values were calculated across the four alpha-diversity indices separately from the 12 physicochemical variables.

Table S2B. Adjusted *P*-values for post hoc pairwise comparisons of alpha-diversity indices.

Variable	Post hoc	Win–Spr	Win–Sum	Win–Aut	Spr–Sum	Spr–Aut	Sum–Aut
Richness	Tukey HSD	0.6126	<0.0001	0.0769	0.0006	0.5207	0.0099
Shannon	Tukey HSD	0.9055	0.0198	1.0000	0.0051	0.8880	0.0217
Simpson	Dunn-BH	0.5040	0.2422	0.7893	0.0720	0.5846	0.2075
Pielou	Tukey HSD	0.6220	0.2372	0.8659	0.0258	0.9685	0.0619

For ANOVA, Tukey HSD adjusted *P*-values are shown; for Kruskal–Wallis, Dunn-BH adjusted *P*-values are shown. Significance groups annotated as “ab” in Figures 2 and 3 share the letters “a” and “b” with the respective groups; groups with a common letter are not significantly different, whereas groups sharing no letter differ at $P < 0.05$. The letter “ab” does not denote a median or intermediate value.

Higher-rank taxonomic comparison (Figure 4)

Relative sequence abundance at the Division level was compared with the 25 genera selected in the original Figure 4B. This analysis evaluates how major sequence-abundance patterns depend on taxonomic resolution. It does not correct primer amplification bias or differences in rDNA copy number.

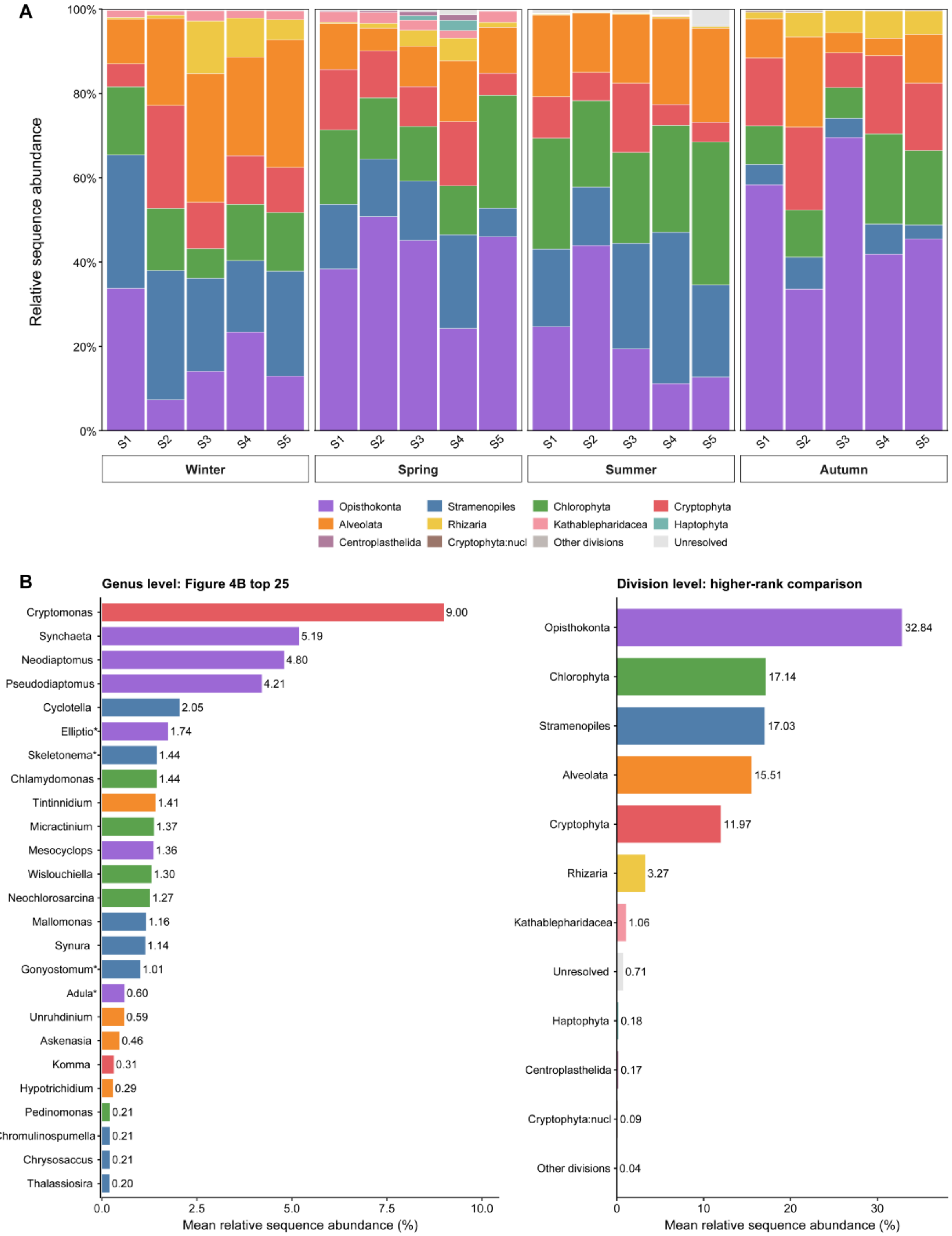

Figure S1. Comparison of aquatic eukaryotic community composition at the Division and genus levels. (A) Relative sequence abundance (%) of principal Divisions in 20 samples, grouped by sampling season (five sites per season). Minor and unresolved assignments are retained in the denominators. (B) Overall mean relative sequence abundance (%) of the top 25 genera used in Figure 4B (left) and principal Divisions (right). The panels have separate x-axis scales; higher-rank totals include sequences that could not be reliably assigned to genus. Relative sequence abundance should not be interpreted as organismal biomass or cell abundance. Genus-level taxonomic assignments were based on the PR2

database. Asterisks (*) indicate genera whose selected representative ASVs showed discrepancies between PR2 assignments and the best named matches obtained from NCBI BLASTn.

Sensitivity analyses excluding Metazoa

To assess the extent to which metazoan sequences influence the study conclusions, all core analyses were repeated after removing confidently assigned Metazoa ASVs. The same 20 samples collected at five fixed sites across four sampling seasons were analyzed.

Supplementary methods

Metazoa exclusion. Taxonomically assigned ASVs labelled Opisthokonta–Metazoa with classification confidence ≥0.70 were removed from the original ASV matrices. ASVs with uncertain assignments were retained. The original rarefied matrix contained 4,903 ASV rows, of which 4,884 had non-zero counts across at least one sample. Removing 319 confidently assigned Metazoa ASVs left 4,565 detected ASVs.

Alpha diversity. Because Metazoa exclusion reduced sample-specific sequencing depths, both datasets were subsampled to 18,156 reads per sample, the minimum remaining depth. A total of 150 rarefaction iterations were used, and their mean index values were retained for each of the 20 biological samples. ASV richness, Shannon diversity, Simpson diversity, and Pielou evenness were summarized across the five sites in each season. Seasonal differences were tested using one-way ANOVA when residual normality (Shapiro–Wilk test) and homogeneity of variance (median-centered Levene test) criteria were met, and Kruskal–Wallis tests otherwise; statistical tests were based on the 20 sample-level mean index values, not on 150 subsampling iterations treated as independent observations (Table S5). Direct full-versus-Non-Metazoa comparisons used paired, two-sided Wilcoxon signed-rank tests on the 20 matched samples; Benjamini–Hochberg adjustment was applied across the four indices (Table S4).

Community composition. PCoA used the original ASV counts after retaining ASVs present in at least two samples with a total of at least 20 reads. A pseudocount of 0.5 was added before centered log-ratio transformation; Euclidean distance in CLR space (Aitchison distance) was used for ordination. Season and site effects were assessed with PERMANOVA using 9,999 permutations with site-restricted seasonal permutation. Homogeneity of within-season dispersion was assessed with PERMDISP.

Beta-diversity partitioning. For each of the six pairwise seasonal comparisons, corresponding samples from the same five sites were compared using presence–absence data. Sørensen dissimilarity (βSOR) was partitioned into turnover (βSIM) and nestedness-resultant (βSNE) components. The primary comparison used the original 58,954-read rarefied matrix before and after Metazoa removal, without treating unequal post-exclusion read depths as equal. A secondary check applied matched-depth subsampling to 18,156 reads, averaged over 150 iterations.

Alpha-diversity sensitivity analysis

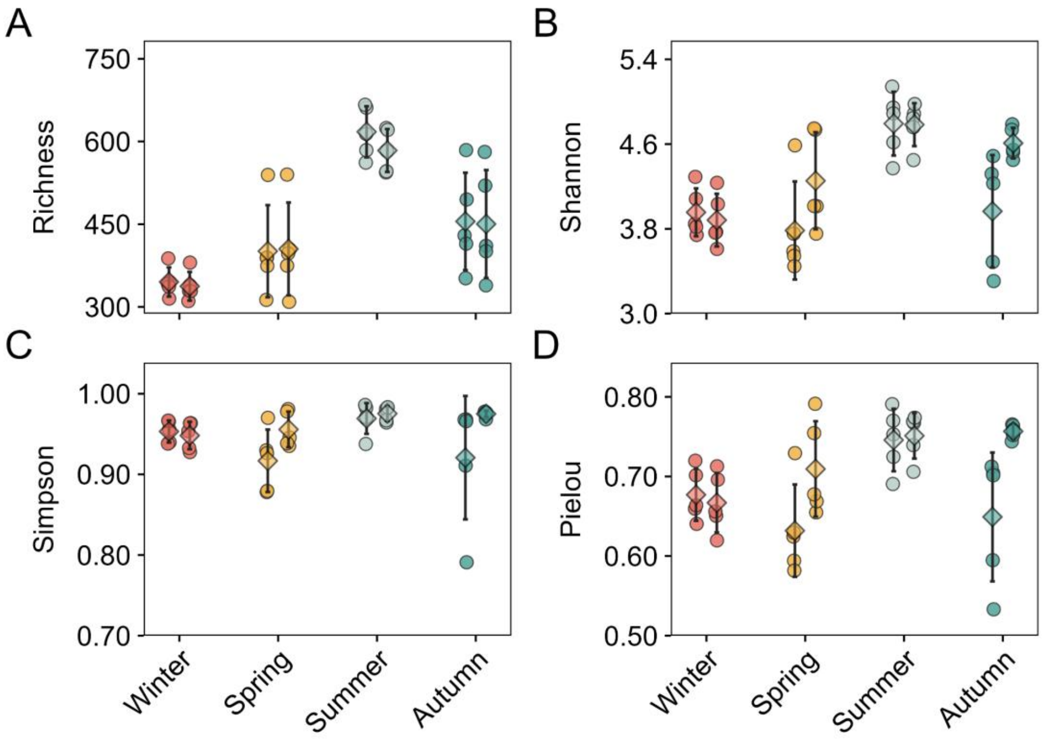

Figure S2. Comparison of alpha-diversity indices for the full community and the community excluding Metazoa: (A) ASV richness, (B) Shannon, (C) Simpson, and (D) Pielou evenness. Colored circular markers represent the five sampling sites in each season; diamonds indicate means and

vertical lines show between-site standard deviations (n = 5). Both datasets were subsampled to 18,156 reads per sample, with 150 subsampling iterations used to estimate each sample-level index. Statistical results are provided in Tables S3–S5. No significance letters or *P*-values are superimposed on the figure.

Table S3. Seasonal alpha-diversity estimates for the full and Non-Metazoa communities at matched sequencing depth.

Community	Season	Richness	Shannon	Simpson	Pielou
Full community	Winter	345.2 ± 26.5	3.956 ± 0.226	0.953 ± 0.014	0.677 ± 0.033
Full community	Spring	401.0 ± 83.6	3.785 ± 0.463	0.917 ± 0.039	0.632 ± 0.058
Full community	Summer	617.6 ± 46.3	4.793 ± 0.301	0.969 ± 0.019	0.746 ± 0.039
Full community	Autumn	455.1 ± 88.4	3.966 ± 0.531	0.921 ± 0.077	0.649 ± 0.081
Non-Metazoa	Winter	337.4 ± 26.1	3.883 ± 0.249	0.948 ± 0.017	0.667 ± 0.037
Non-Metazoa	Spring	405.0 ± 84.2	4.253 ± 0.458	0.956 ± 0.022	0.709 ± 0.060
Non-Metazoa	Summer	583.7 ± 38.9	4.783 ± 0.203	0.975 ± 0.009	0.751 ± 0.029
Non-Metazoa	Autumn	450.3 ± 97.9	4.609 ± 0.145	0.975 ± 0.004	0.757 ± 0.009

Note. Values are mean ± SD across the five sites per season. Estimates for each sample were averaged over 150 random subsampling iterations at 18,156 reads. ASV richness denotes observed ASV number.

Table S4. Paired comparisons of alpha-diversity indices before and after Metazoa exclusion (20 matched samples).

Metric	Full mean	Non-Metazoa mean	Mean change	Wilcoxon W	*P*	BH-adjusted *P*
Richness	454.7	444.1	-10.6	40.0	0.0136	0.0400
Shannon	4.125	4.382	+0.257	48.0	0.0328	0.0400
Simpson	0.940	0.964	+0.024	50.0	0.0400	0.0400
Pielou	0.676	0.721	+0.045	45.0	0.0240	0.0400

Note. Two-sided paired Wilcoxon signed-rank tests were applied to 20 sample-level index estimates (not 150 subsampling replicates). Mean change is Non-Metazoa minus full community. Benjamini–Hochberg (BH) adjustment was applied across the four indices.

Table S5. Seasonal omnibus tests of alpha-diversity indices before and after Metazoa exclusion at matched sequencing depth.

Community	Index	Omnibus test and statistic	Overall *P*	Highest mean season
Full	Richness	ANOVA: F = 15.647 (3,16)	0.0001	Summer
Full	Shannon	ANOVA: F = 6.440 (3,16)	0.0046	Summer
Full	Simpson	Kruskal-Wallis: H = 6.749 (3)	0.0804	Summer
Full	Pielou	ANOVA: F = 4.012 (3,16)	0.0263	Summer
Non-Metazoa	Richness	ANOVA: F = 11.453 (3,16)	0.0003	Summer
Non-Metazoa	Shannon	ANOVA: F = 9.565 (3,16)	0.0007	Summer
Non-Metazoa	Simpson	ANOVA: F = 4.362 (3,16)	0.0200	Summer

Community	Index	Omnibus test and statistic	Overall *P*	Highest mean season
Non-Metazoa	Pielou	ANOVA: F = 5.925 (3,16)	0.0064	Autumn

Note. For each community, Shapiro-Wilk tests of ANOVA residuals and median-centered Levene tests were used to select either one-way ANOVA (F; df = 3, 16) or Kruskal-Wallis (H; df = 3). Overall P-values are unadjusted. Each seasonal mean is based on five biological samples; 150 equal-depth subsampling iterations were averaged within each sample before statistical testing.

Community composition and dispersion sensitivity analysis

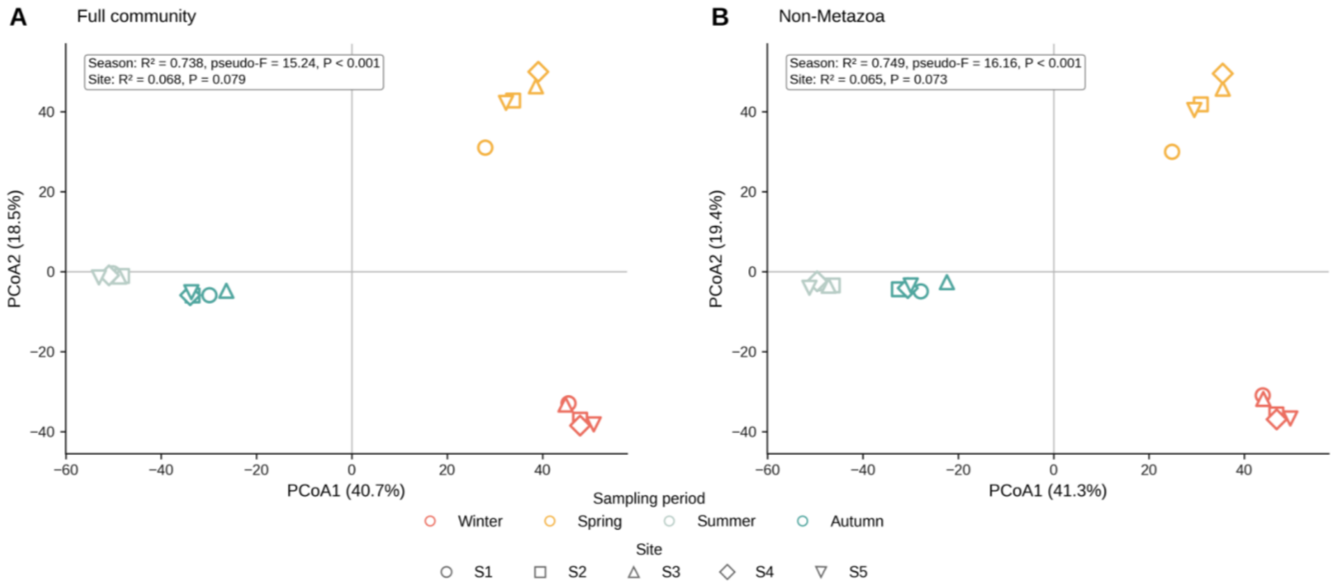

Figure S3. Aitchison principal coordinates analysis (PCoA) of the full community (A) and the Non-Metazoa community (B). Colors identify sampling seasons and open shapes identify the five fixed sampling sites. Axis percentages describe the variance represented by each coordinate in the corresponding dataset. Site-adjusted PERMANOVA results are summarized in Table S6.

Table S6. Seasonal and site effects on community composition before and after Metazoa exclusion (PERMANOVA).

Community	Factor	R^2	Pseudo-F	*P*
Full community	Season	0.738	15.24	<0.001
Full community	Site	0.068	1.05	0.076
Non-Metazoa	Season	0.749	16.16	<0.001
Non-Metazoa	Site	0.065	1.05	0.0730

Note. ASVs in the raw-count matrices were filtered using the original analysis thresholds, followed by a 0.5 pseudocount, centered log-ratio transformation, and Euclidean (Aitchison) distances. Permutation P-values were based on 9,999 permutations; seasonal tests used site-restricted permutations. R^2 represents the fraction of total community variation attributable to the tested factor.

Table S7. Multivariate dispersion among seasonal groups before and after Metazoa exclusion (PERMDISP).

Community	F	*P*	Winter distance	Spring distance	Summer distance	Autumn distance
Full community	6.457	0.0150	28.83	32.43	37.05	34.51
Non-Metazoa	3.470	0.0508	27.91	30.77	34.22	30.34

Note. Mean values represent distance to the seasonal centroid in the Aitchison ordination space. Permutation-based *P*-values are based on 9,999 permutations and may vary slightly with the random seed. The Non-Metazoa result (*P* = 0.0508) is close to 0.05 and should be interpreted cautiously.

Beta-diversity partitioning sensitivity analysis

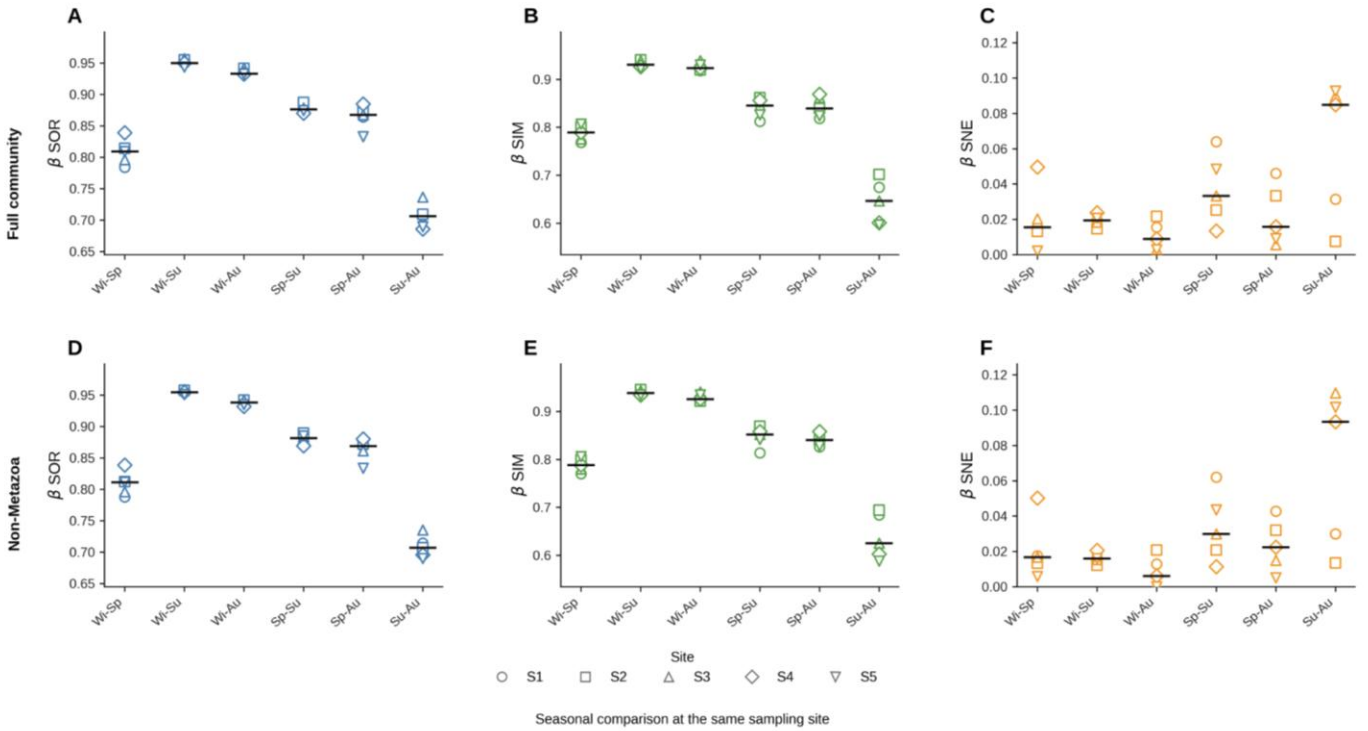

Figure S4. Beta-diversity partitioning for the full community (A–C) and Non-Metazoa community (D–F), based on five same-site seasonal pairs per comparison. Blue, green and orange indicate Sørensen dissimilarity (βSOR), Simpson turnover (βSIM) and nestedness-resultant dissimilarity (βSNE), respectively. Symbols denote sites and black horizontal lines denote medians. The underlying primary pairwise results are presented in Table S8; a depth-controlled check is summarized in Table S9.

Table S8. Seasonal beta-diversity partitioning before and after Metazoa removal using the original rarefied dataset.

Community	Season comparison	Median βSOR	Median βSIM	Median βSNE
Full community	Winter–Spring	0.809	0.789	0.016
Full community	Winter–Summer	0.950	0.931	0.019
Full community	Winter–Autumn	0.933	0.924	0.009
Full community	Spring–Summer	0.876	0.845	0.033
Full community	Spring–Autumn	0.868	0.839	0.016
Full community	Summer–Autumn	0.706	0.647	0.085
Non-Metazoa	Winter–Spring	0.811	0.788	0.017
Non-Metazoa	Winter–Summer	0.955	0.939	0.016
Non-Metazoa	Winter–Autumn	0.938	0.926	0.006
Non-Metazoa	Spring–Summer	0.882	0.852	0.030
Non-Metazoa	Spring–Autumn	0.869	0.840	0.022
Non-Metazoa	Summer–Autumn	0.707	0.625	0.093

Note. Values are medians of five corresponding-site comparisons. ASV occurrences were based on the original matrix rarefied to 58,954 reads per sample, followed by Metazoa removal without treating the resulting sequence depths as equal. The identity βSOR = βSIM + βSNE applies to individual paired observations; medians of each component need not sum exactly.

Table S9. Matched-depth check of seasonal beta-diversity partitioning before and after Metazoa removal.

Community	Season comparison	Median βSOR	Median βSIM	Median βSNE

Community	Season comparison	Median βSOR	Median βSIM	Median βSNE
Full community	Winter–Spring	0.796	0.769	0.014
Full community	Winter–Summer	0.948	0.928	0.021
Full community	Winter–Autumn	0.928	0.917	0.010
Full community	Spring–Summer	0.864	0.831	0.034
Full community	Spring–Autumn	0.859	0.825	0.016
Full community	Summer–Autumn	0.679	0.618	0.092
Non-Metazoa	Winter–Spring	0.803	0.771	0.022
Non-Metazoa	Winter–Summer	0.953	0.936	0.017
Non-Metazoa	Winter–Autumn	0.936	0.920	0.009
Non-Metazoa	Spring–Summer	0.872	0.847	0.024
Non-Metazoa	Spring–Autumn	0.866	0.837	0.020
Non-Metazoa	Summer–Autumn	0.694	0.633	0.084

Note. Secondary check at a common depth of 18,156 reads per sample using 150 subsampling iterations. Values summarize five same-site seasonal comparisons. Because medians are calculated separately for each component, their totals may differ slightly from the separately calculated βSOR median.

Table S10. NCBI BLASTn cross-check of 25 representative 18S V4 ASVs from the 25 PR2-annotated genera shown in the original Figure 4B and Figure 5B

ASV ID	PR2 genus	NCBI BLASTn match(es)	GenBank accession(s)	Identity (%)	Query cover (%)	Genus-level assessment
ASV3	*Cryptomonas*	*Cryptomonas curvata*	KF907375	100.00	100.00	Concordant named best match
ASV1	*Synchaeta*	*Synchaeta pectinata*	KP875584	100.00	100.00	Concordant named best match
ASV2	*Neodiaptomus*	*Neodiaptomus schmackeri*	KR048710	100.00	100.00	Concordant named best match
ASV7	*Pseudodiaptomus*	*Pseudodiaptomus inopinus*	GU969194	97.12	100.00	Concordant named best match
ASV17	*Cyclotella*	*Uncultured stramenopile (best); Cyclotella atomus (lower score)*	EU143915; MZ798444	99.68	100.00	Best hit unnamed; named PR2 match lower-ranked
ASV9	*Elliptio**	*Aculamprotula polysticta; Solenaia carinata; Lampsilis siliquoidea; Unio pictorum*	MK687414; MK683000; KY978476; KC429349	100.00	100.00	**Discordant named best match**
ASV30	*Skeletonema*	*Skeletonema potamos*	OP698009	100.00	100.00	Concordant named best match
ASV81	*Chlamydomonas*	*Chlamydomonas monadina; Microglena coccifera*	FR854379; PP147735	100.00	100.00	Intergeneric co-best match
ASV34	*Tintinnidium*	*Tintinnidium sp.*	JN831803	100.00	100.00	Concordant named best match
ASV23	*Micractinium*	*Chlorella sorokiniana; Micractinium* sp.	PX503755; PV434772	100.00	100.00	Intergeneric co-best match
ASV24	*Mesocyclops*	*Microcyclops varicans; Mesocyclops cf. chaci*	MK106114; MK370203	98.41	100.00	Intergeneric co-best match
ASV52	*Wislouchiella*	*Uncultured Chlorophyta (best); Wislouchiella planctonica (lower score)*	JX296631; AF252547	99.01	100.00	Best hit unnamed; named PR2 match lower-ranked
ASV40	*Neochlorosarcina*	*Neochlorosarcina negevensis*	LC425385	98.35	100.00	Concordant named best match
ASV65	*Mallomonas*	*Mallomonas crassisquama*	PQ008730	99.03	100.00	Concordant named best match
ASV19	*Synura*	*Synura glabra*	PQ039828	100.00	100.00	Concordant named best match
ASV27	*Gonyostomum**	*Merotricha bacillata*	AB512124	99.05	100.00	**Discordant named best match**
ASV36	*Adula**	*Sinomytilus harmandi; Limnoperna fortunei*	MK629443; MG674670	100.00	100.00	**Discordant named best match**
ASV39	*Unruhdinium*	*Unruhdinium minimum*	JQ639767	100.00	100.00	Concordant named best match
ASV97	*Askenasia**	*Balanion planctonicum*	PQ775545	100.00	100.00	**Discordant named best match**
ASV72	*Komma*	*Komma caudata*	AM901358	100.00	100.00	Concordant named best match
ASV99	*Hypotrichidium*	*Hypotrichidium bichromum*	PP906280	100.00	100.00	Concordant named best match
ASV150	*Pedinomonas*	*Pedinomonas minor*	MW471015	99.33	100.00	Concordant named best match
ASV183	*Chromulinospumella*	*Uncultured alga*	KC631669	99.03	100.00	No named genus-level support
ASV89	*Chrysosaccus*	*Chrysosaccus* sp.	EF165120	97.73	100.00	Concordant named best match
ASV134	*Thalassiosira*	*Thalassiosira gessneri*	OM899758	100.00	100.00	Concordant named best match

Note. One most-abundant ASV was selected from each of the 25 PR2-assigned genera in the original Figure 4B. These genera include the 17 original Figure 5B seasonal indicator genera. Taxonomic assignments displayed in Figures 4B and 5B remain PR2-based. Asterisks (*) indicate discrepancies between PR2 genus assignments and the best named NCBI BLASTn matches.
